# Modeling resource conflict and inequality in fragile systems: A spatial agent-based framework with a Uganda case study

**DOI:** 10.1371/journal.pone.0357283

**Published:** 2026-09-16

**Authors:** Ronald Katende

**Affiliations:** Department of Mathematics, Kabale University, Kikungiri Hill, Kabale, Uganda; University of Salerno: Universita degli Studi di Salerno, ITALY

## Abstract

This study develops a spatial agent-based framework for examining how unequal resource access, environmental stress, institutional support, movement, and adaptive cooperation jointly shape conflict pressure and resource inequality. A Uganda case study initializes spatial heterogeneity from WorldPop 2020 population data and CHIRPS v2.0 October–November 2025 rainfall. The primary experiment uses an 80×80 grid, 250 agents, 120 time steps, and 20 baseline seeds. Parameter dependence is evaluated using 128 Latin-hypercube designs with five stochastic replicates per design, time-aware cluster-robust regressions, partial rank correlations, dense one-at-a-time sweeps, structural ablations, and grid/agent-count robustness checks. In the baseline ensemble, the Gini coefficient rises from 0.192 to 0.868 while mean cooperation falls from 0.547 to 0.118; conflict pressure stabilizes near 0.207 after an early transient. Institutional support increases cooperation and mean resources and reduces inequality over the tested range, while also altering the interaction-level conflict index, showing that lower modeled conflict is not necessarily a better system state. Removing the resource-disparity term reduces but does not eliminate conflict, and suppressing behavioral adaptation raises final conflict by about 40% relative to the baseline. The framework is therefore best interpreted as a transparent mechanism-exploration and stress-testing tool rather than an event-forecasting model.

## 1. Introduction

Resource conflicts in socio-ecological systems remain a serious development challenge in many low-income settings. They are especially difficult where pressure on land, water, and grazing resources interacts with weak institutions, unequal access, and contested local authority. In Sub-Saharan Africa, such conflicts are rarely explained by scarcity alone. They are also shaped by power relations, land tenure arrangements, exclusion, mobility, and the capacity of formal and informal institutions to manage competing claims [1–4]. Policy analysis in these settings therefore requires tools that can represent both environmental pressure and the decentralized decisions through which conflict, cooperation, and inequality emerge.

Computational modeling provides one way to study these coupled dynamics. Agent-based models are useful when outcomes depend on heterogeneous actors, local interaction, adaptation, and feedback between behavior and environmental conditions [5–7]. System-dynamics models are useful when the main concern is aggregate feedback, delayed effects, and long-run sustainability trade-offs [8]. Spatially explicit and hybrid models are useful when resource access and conflict exposure depend on location, neighborhood structure, and landscape heterogeneity [9,10]. Each approach contributes useful insight, but each also has limits when used alone for resource-conflict analysis in fragile and data-constrained contexts.

Agent-based models are particularly relevant for resource governance because conflict often arises through repeated local encounters over access, enforcement, movement, and response to shocks. They can represent actors with different resources, incentives, and behavioral rules, while tracking how macro-level outcomes such as inequality, cooperation breakdown, or conflict concentration emerge from micro-level behavior [5,7]. This flexibility explains their use in land-use change, common-pool resource management, and coupled human-environment systems [9,10]. However, the same flexibility creates risks. Without transparent assumptions, behavioral rules, parameter choices, and validation checks, an ABM can generate plausible patterns without providing credible evidence for interpretation or policy use [6,7].

System-dynamics and spatially explicit models address related aspects of the problem. System-dynamics models clarify feedback loops, accumulations, delays, degradation, regeneration, and long-run policy effects [8]. Spatial models show why location is not merely a background feature. Terrain quality, water access, settlement structure, mobility corridors, and neighborhood effects can shape both resource use and conflict exposure [9]. Recent work linking agent-based modeling to food, energy, and water systems further shows the value of modeling human decisions and environmental processes together across scales [10]. Still, many existing models are better suited to land-use change or resource allocation than to conflict as a dynamic outcome of inequality, ecological stress, institutional conditions, and adaptive behavior.

Three limitations are especially important for the present study. First, behavioral heterogeneity is often simplified, even though differences in endowments, incentives, social position, and learning can materially affect collective outcomes [5,11,12]. Second, two-way feedback between human action and environmental change is unevenly represented. In many applications, the environment constrains behavior, but ecological change is not modeled as an endogenous consequence of extraction, mobility, or conflict itself [6,10,13]. Third, adaptation is often represented weakly, although learning, imitation, and strategy revision are central to whether cooperation stabilizes or collapses over time [14,15]. These issues matter in fragile settings because conflict rarely follows from a single driver. It emerges from interaction among unequal access, environmental pressure, local institutions, and adaptive behavior.

This study develops a spatially explicit agent-based framework for examining how resource inequality, environmental stress, institutional support, movement, and adaptive interaction shape conflict and cooperation in fragile resource systems. The aim is not to forecast conflict events. Instead, the model makes the feedback structure explicit so that the effects of power asymmetry, value differences, resource disparities, environmental stress, cooperation, degradation, recovery, and movement can be tested separately and jointly.

The contribution is methodological and interpretive rather than predictive. In addition to reporting the agent-level rules, the analysis jointly varies the continuous parameters, estimates independent and time-varying effects, decomposes the conflict index, removes individual mechanisms through structural ablation, and checks sensitivity to numerical resolution. These analyses distinguish relationships imposed directly by the model definition from relationships that arise through endogenous feedback among resources, cooperation, movement, and environmental quality. This is important because lower measured conflict can coexist with high inequality, declining cooperation, or depleted resources [6,7,11].

The empirical case study focuses on Uganda. Spatial population heterogeneity is initialized from WorldPop and environmental heterogeneity from CHIRPS rainfall, while the resulting conflict surface remains a model output rather than a forecast of observed violent events. The framework is therefore intended for mechanism exploration, sensitivity analysis, and transparent policy stress testing in data-constrained settings [8,10].

## 2. Materials and methods

The model is a spatial agent-based mechanism-exploration framework rather than an event-forecasting system. It is designed to test how unequal resource access, environmental stress, institutional support, movement, and adaptive cooperation interact over time. The analysis assesses parameter transparency, independent parameter effects, temporal dependence, and whether the reported conflict dynamics are mechanically imposed by any single term in the conflict index. The model rules are stated explicitly, and the evaluation combines global sensitivity, time-aware regression, rank-based sensitivity, one-at-a-time response sweeps, structural ablation, and resolution checks [16,17].

### 2.1. Ethics statement

This study did not involve human participants, animal subjects, patient-level records, interviews, surveys, or private personal data. The empirical component used publicly available population and rainfall datasets together with simulated model outputs. Therefore, institutional ethics approval and informed consent were not required.

### 2.2. Entities, state variables, and spatial setting

The model contains *N* agents on a two-dimensional grid of size W×H. At time *t*, agent *i* has location $X_{i}(t)=(x_{i}(t),y_{i}(t))$, power $P_{i}(t)$, value orienta*t*ion $V_{i}(t)$, resource holdings $R_{i}(t)$, and cooperation propensity $C_{i}(t)$. The variables $P_{i}(t)$, $V_{i}(t)$, and $C_{i}(t)$ are normalized to [0,1], while $R_{i}(t)\geq 0$. Each grid cell has terrain quality Q(x,y,t)∈[0,1] and environmental stress E(x,y,t)∈[0,1]. These are model-scale quantities rather than fitted empirical coefficients. Heterogeneity is retained because actor differences and spatial conditions can materially affect aggregate outcomes in social–ecological ABMs [5,11,12].

Agents interact locally. Let ${𝒩}_{i}(t)$ be the interaction neighborhood of agent *i*. Local interaction is used because competition, cooperation, movement, and resource access are spatially mediated processes [9,10]. Each simulation cycle applies the same order: movement or local occupancy, pair interaction, conflict/cooperation calculation, resource update, cooperation update, and environmental update.

### 2.3. Conflict pressure and cooperative gain

For interacting agents *i* and *j*, the pairwise disparity terms are


$$\begin{array}{c} d_{ij}^{P}(t)=|P_{i}(t)-P_{j}(t)|, \end{array}$$ (1)

$$\begin{array}{c} d_{ij}^{V}(t)=|V_{i}(t)-V_{j}(t)|, \end{array}$$ (2)

$$\begin{array}{c} d_{ij}^{R}(t)=|R_{i}(t)-R_{j}(t)|. \end{array}$$ (3)

The environmental term is the mean stress experienced by the interacting pair,


$$\begin{array}{c} E_{ij}(t)=\frac{1}{2}\left[E(X_{i}(t),t)+E(X_{j}(t),t)\right]. \end{array}$$ (4)

Conflict pressure is then


$$\begin{array}{c} S_{ij}(t)=\alpha d_{ij}^{P}(t)+\beta d_{ij}^{V}(t)+\gamma d_{ij}^{R}(t)+\delta E_{ij}(t), \end{array}$$ (5)

where α,β,γ,δ≥0. Equation (5) is an additive mechanism index, not a universal law of conflict and not a count of observed violent events. Its four components represent mechanisms repeatedly emphasized in work on land conflict, common-pool governance, pastoral stress, and farmer–herder conflict [1,3,4].

Cooperative gain is modeled separately:


$$\begin{array}{c} G_{i}(t)=\kappa C_{i}(t)C_{j}(t), \end{array}$$ (6)

with the same form for $G_{j}(t)$. The parameter κ represents contextual support for cooperation, including mediation, credible enforcement, or institutional conditions that make cooperation beneficial [2].

### 2.4. Resource, cooperation, environmental, and movement feedback

Resource holdings evolve as


$$\begin{array}{c} R_{i}(t+1)={\left[R_{i}(t)+G_{i}(t)+\lambda Q(X_{i}(t),t)-S_{ij}(t)-\mu E(X_{i}(t),t)\right]}_{+}, \end{array}$$ (7)

where $\left[z{]}_{+}=\max\{z,0\right\}$. Cooperation adapts according to


$$\begin{array}{c} C_{i}(t+1)=\Pi_{\left[0,1\right]}\left[C_{i}(t)+\rho \frac{G_{i}(t)-S_{ij}(t)}{\bar{R}(t)+\varepsilon }\right], \end{array}$$ (8)

where $\bar{R}(t)=N^{-1}\sum_{k=1}^{N}R_{k}(t)$ and ε>0. Terrain quality changes through recovery and use pressure,


$$\begin{array}{c} Q(x,y,t+1)=\Pi_{\left[0,1\right]}\left[Q(x,y,t)+\eta ℛ(x,y,t)-\zeta D(x,y,t)\right], \end{array}$$ (9)

with


$$\begin{array}{c} D(x,y,t)=\sum_{i=1}^{N}1\{X_{i}(t)=(x,y)\},\;ℛ(x,y,t)=1-E(x,y,t). \end{array}$$ (10)

Movement is probabilistic rather than perfectly optimizing. If $z\in {ℳ}_{i}(t)$ is a candidate cell,


$$\begin{array}{c} \mathrm{Pr}\{X_{i}(t+1)=z\}=\frac{Q(z,t)+\epsilon_{Q}}{\sum_{z^{\prime }\in {ℳ}_{i}(t)}[Q(z^{\prime },t)+\epsilon_{Q}]}. \end{array}$$ (11)

**Fig 1:**
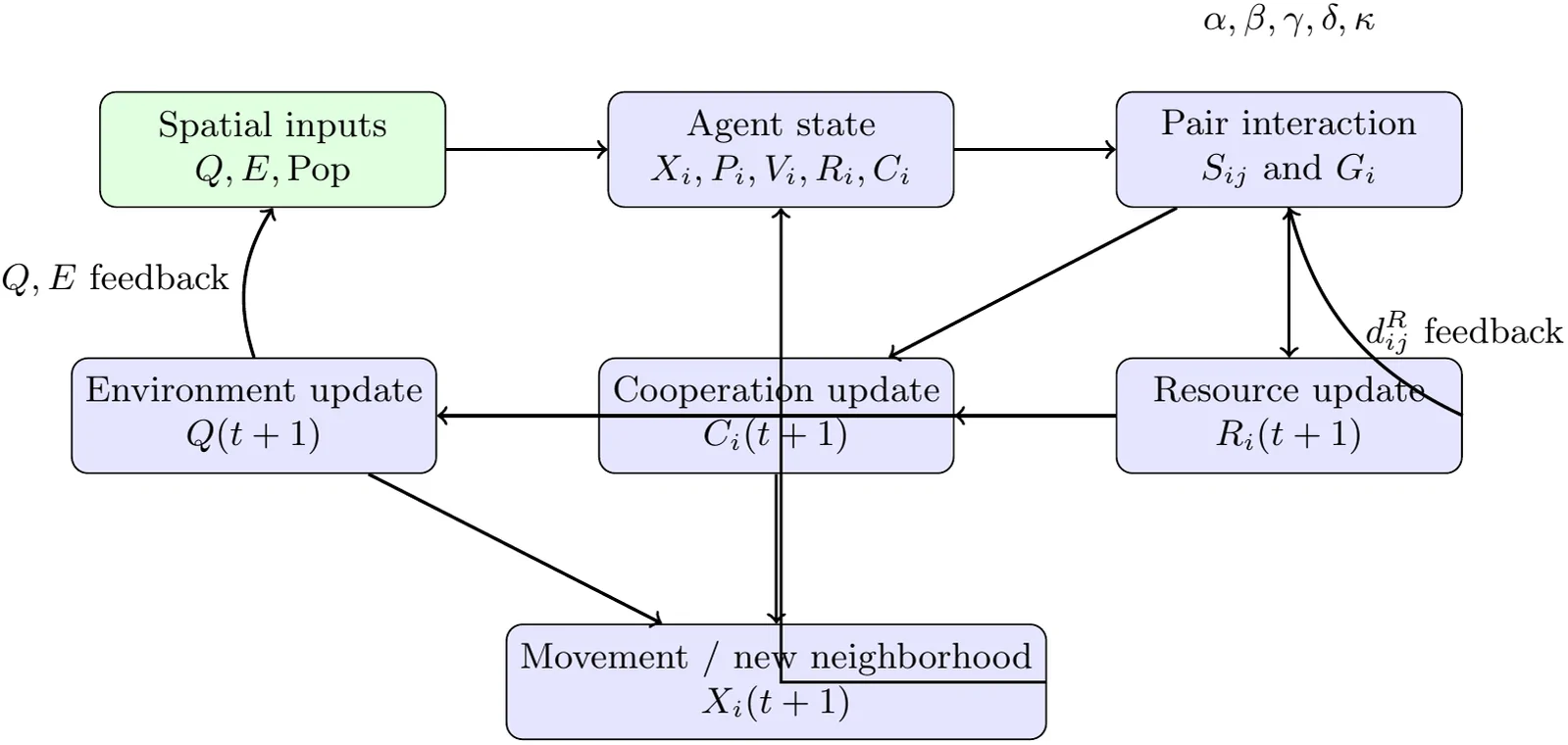
Feedback architecture of the spatial ABM. Spatial inputs initialize heterogeneous agent states. Pair interactions generate conflict pressure and cooperative gain, which feed back through resources, cooperation, environmental quality, and movement. The resource-disparity term therefore evolves endogenously rather than remaining a fixed input.

The feedback architecture implied by Equations (5)–(11) is summarized in Fig 1. Unlike a linear execution-only flowchart, Fig 1 makes the endogenous feedback explicit: conflict and cooperation alter resources; resources alter subsequent conflict disparities and adaptation; environmental quality affects resource gain and movement; and movement changes future neighborhoods and degradation pressure.

### 2.5. Inequality and recorded outputs

Resource inequality is measured by the Gini coefficient


$$\begin{array}{c} 𝒢(t)=\frac{\sum_{i=1}^{N}\sum_{j=1}^{N}|R_{i}(t)-R_{j}(t)|}{2N\sum_{i=1}^{N}R_{i}(t)}. \end{array}$$ (12)

The model records conflict per interaction, total conflict pressure, mean and median resources, resource coefficient of variation, mean cooperation, mean terrain quality, environmental stress, interaction counts, 𝒢(t), and the four separate components of Equation (5). Conflict pressure and 𝒢(t) are outputs with different meanings; neither is treated as a welfare function.

### 2.6. Spatial empirical inputs and initialization

The analysis uses a Uganda-focused spatial domain bounded by longitude $29.5^{\circ }--35.1^{\circ }$E and latitude $1.5^{\circ }$S–$4.3^{\circ }$N. Population heterogeneity is taken from the WorldPop 2020 Uganda 1-km population-count product [18,19]. The offline preprocessing used 243,717 positive WorldPop source cells and yielded an aggregate population of 42,107,362 over the configured grid input. Environmental heterogeneity is taken from the CHIRPS v2.0 October–November 2025 rainfall field [20]. These two inputs are spatially resolved and are used to initialize the model rather than to fit the conflict equation.

Let $\tilde{Pop}(z)$ be normalized population exposure in cell *z*. Agent placement follows


$$\begin{array}{c} \mathrm{Pr}\{X_{i}(0)=z\}=\frac{Pop(z)}{\sum_{z^{\prime }}Pop(z^{\prime })}. \end{array}$$ (13)

If *M*(*z*) is rainfall, the normalized rainfall field is


$$\begin{array}{c} \tilde{M}(z)=\frac{M(z)-\min_{z}M(z)}{\max_{z}M(z)-\min_{z}M(z)},\;E(z)=1-\tilde{M}(z),\;Q(z,0)=\tilde{M}(z). \end{array}$$ (14)

Rainfall is therefore a transparent environmental proxy in the present run, not a claim that precipitation fully measures land quality.

Initial attributes are


$$\begin{array}{c} P_{i}(0)=\Pi_{\left[0,1\right]}\left[0.2+0.8\tilde{Pop}(X_{i}(0))\right], \end{array}$$ (15)

$$\begin{array}{c} V_{i}(0)\sim Uniform(0,1), \end{array}$$ (16)

$$\begin{array}{c} R_{i}(0)={\left[0.1+Q(X_{i}(0),0)+0.25\tilde{Pop}(X_{i}(0))\right]}_{+}, \end{array}$$ (17)

$$\begin{array}{c} C_{i}(0)=\Pi_{\left[0,1\right]}\left[0.5+0.2\{1-E(X_{i}(0))\}-0.1P_{i}(0)\right]. \end{array}$$ (18)

Population density is used here as an exposure-based initialization proxy and should not be interpreted as a direct measure of political power.

### 2.7. Parameter values, sensitivity ranges, and justification

Table 1 reports the baseline settings and the predeclared ranges used in the final global sensitivity analysis. The intervals are robustness ranges around the reference parameterization, not empirical confidence intervals. The mechanisms and expected signs are literature-motivated, while the exact numerical values are not claimed to be field-calibrated. This distinction directly separates parameter transparency from empirical estimation.

**Table 1:** Baseline parameters and predeclared sensitivity ranges used in the analysis. The ranges are robustness intervals, not empirical confidence intervals.

Parameter	Meaning	Baseline	Sensitivity range	Model role
α	Power-disparity weight	0.30	0.150–0.450	Conflict pressure
β	Value-orientation-difference weight	0.20	0.100–0.300	Conflict pressure
γ	Resource-disparity weight	0.35	0.175–0.525	Conflict pressure
δ	Environmental-stress weight	0.15	0.075–0.225	Conflict pressure
κ	Institutional support for cooperation	0.75	0.375–1.125	Cooperative gain
λ	Terrain contribution to resources	0.08	0.040–0.120	Resource update
μ	Environmental resource-loss scale	0.06	0.030–0.090	Resource update
η	Terrain recovery rate	0.01	0.005–0.020	Environmental feedback
ζ	Terrain degradation coefficient	0.03	0.015–0.060	Environmental feedback
ρ	Cooperation adaptation rate	0.04	0.020–0.080	Behavioral adaptation
$p_{m}$	Movement probability	0.30	0.150–0.450	Spatial adaptation
*r*	Interaction radius	1	fixed	Local interaction

The numerical baselines in Table 1 are reference settings rather than estimates fitted to observed conflict. The four conflict weights sum to one at baseline (α+β+γ+δ=1), which keeps the additive index on an interpretable relative scale while assigning slightly greater reference weight to resource and power disparities than to value orientation and environmental stress. The remaining baselines were chosen to keep cooperative gain, resource gain/loss, recovery, degradation, adaptation, and movement on comparable bounded per-step scales over the 120-step horizon. Because these numerical choices are not empirically identified, the sensitivity analysis deliberately perturbs the principal parameters over broad predeclared intervals; most are varied from 50% below to 50% above baseline, while the smaller recovery, degradation, and adaptation rates are varied over similarly broad positive ranges. The literature supports the mechanisms and expected directions, not the exact numerical values [1–3,10,13].

### 2.8. Simulation and sensitivity design

The primary experiment uses an 80×80 model grid, 250 agents, and 120 time steps. Baseline dynamics are averaged over 20 seeds (1729–1748). Global sensitivity uses 128 Latin-hypercube designs with five stochastic replicates per design (640 runs). The complete trajectories are sampled at 13 time points per run, giving 8,320 observations for each time-aware regression. One-at-a-time sweeps vary κ, γ, δ, ρ, and ζ over 25 values with five seeds each. Structural ablation uses seven model variants and five seeds. Resolution robustness uses four grid/agent configurations and five seeds. Table 2 summarizes the design.

**Table 2:** Analysis design used to assess sensitivity, temporal dependence, structural dependence, and numerical robustness.

Component	Specification
Spatial domain	Uganda-focused bounds: 29.5–35.1°E, 1.5°S–4.3°N
Baseline model	80×80 grid; 250 agents; 120 time steps
Baseline stochastic replication	20 seeds (1729–1748)
Global sensitivity	128 Latin-hypercube designs × 5 seeds = 640 runs
Time-aware panel data	13 recorded times/run; 8,320 observations per outcome
One-at-a-time diagnostics	5 parameters × 25 values × 5 seeds
Structural ablation	7 variants × 5 seeds
Resolution robustness	(40,125), (60,250), (80,250), (80,500); 5 seeds each
Spatial inputs	WorldPop Uganda 2020; CHIRPS Oct–Nov 2025
Primary outputs	Conflict/interact., Gini, cooperation, mean resources

### 2.9. Statistical analysis of independent and time-varying effects

To estimate independent parameter effects, each swept parameter is standardized within the joint Latin-hypercube ensemble. For outcome $Y_{rt}$ in simulation run *r* at normalized time $\tau_{t}\in [0,1]$, the fitted model is


$$\begin{array}{c} Y_{rt}=b_{0}+\sum_{k}b_{k}Z_{rk}+b_{t}\tau_{t}+b_{t^{2}}\tau_{t}^{2}+\sum_{k}c_{k}Z_{rk}\tau_{t}+\varepsilon_{rt}, \end{array}$$ (19)

where $Z_{rk}$ is the standardized value of parameter *k*. Ordinary least squares is used with covariance clustered by simulation run, so repeated observations from the same stochastic run are not treated as independent. The $b_{k}$ coefficients measure conditional main effects in the jointly varied ensemble; the $c_{k}$ coefficients test whether parameter influence changes over time.

As a nonparametric complement, partial rank correlation coefficients (PRCCs) are computed at the final simulation step. Parameter and outcome values are rank transformed; for each parameter, the ranks of that parameter and the outcome are separately residualized against all other swept parameters, and the Pearson correlation of the two residual series is reported. PRCC therefore provides a monotonic global-sensitivity diagnostic that does not depend on the scale of the original parameters.

To investigate nonlinear response and possible thresholds without overclaiming formal bifurcations, one-at-a-time curves are evaluated for κ, γ, δ, ρ, and ζ. The candidate transition point is defined as the sampled parameter value with the largest absolute finite-difference slope. These are reported as steep-response locations only, not as mathematically proven tipping points.

### 2.10. Structural validation and interpretation scope

Three additional checks separate model structure from emergent feedback. First, conflict is decomposed into the four additive contributions in Equation (5). Second, each conflict component is removed separately, together with separate no-adaptation and no-movement controls. Third, the simulation is repeated under alternative grid/agent resolutions. These tests address the possibility that the reported conflict pattern is simply an algebraic consequence of one chosen term.

The Uganda run is spatially resolved with observed population and rainfall inputs, but no observed georeferenced conflict-event surface is matched to the October–November 2025 environmental window. Accordingly, the modeled conflict surface is reported as a spatial model diagnostic rather than as observed-event validation, and no administrative-unit comparison is used to claim predictive spatial validity.

## 3. Results

### 3.1. Spatial forcing and baseline dynamics

**Fig 2:**
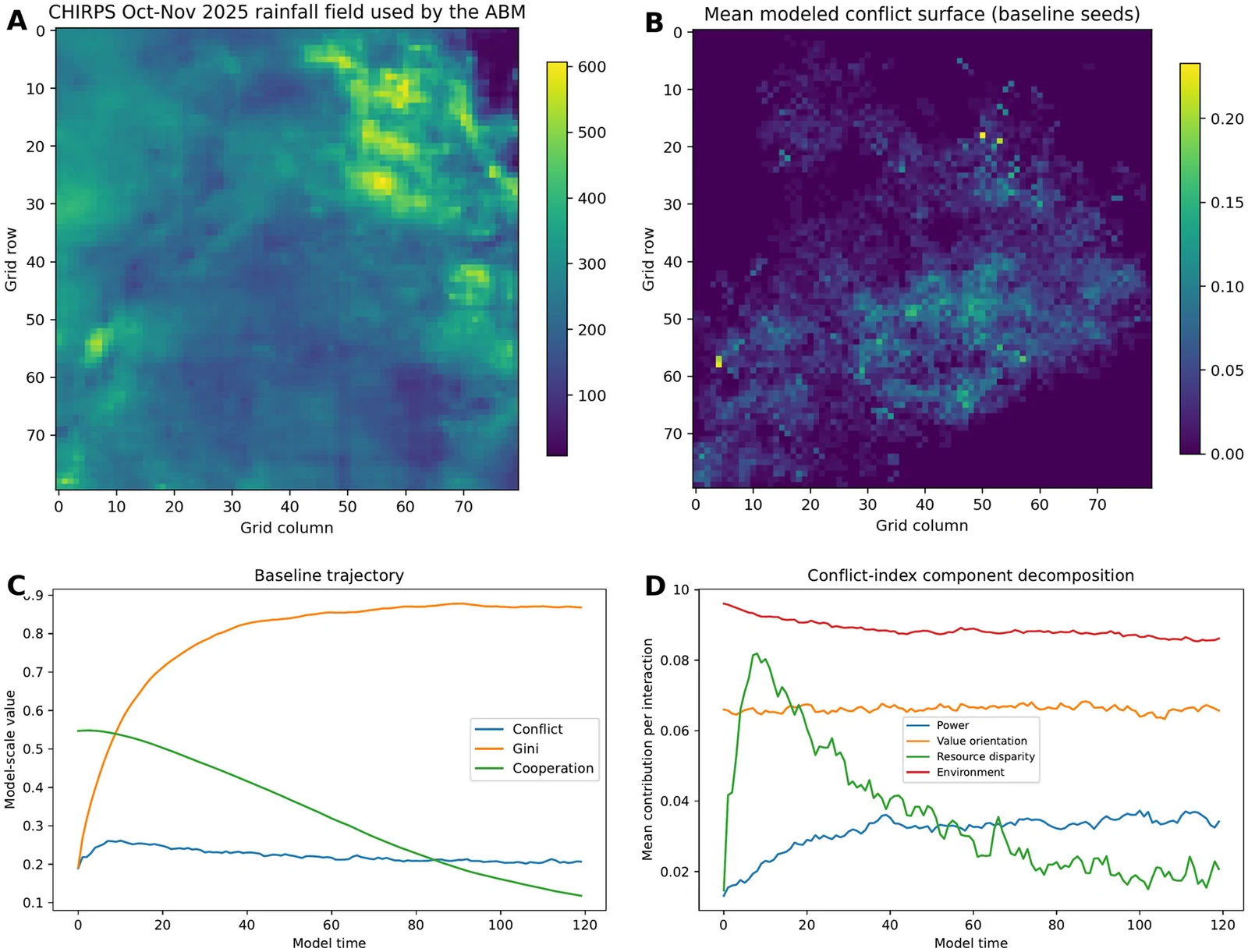
Spatial forcing and baseline dynamics for the Uganda analysis. Panel (A) shows the observed CHIRPS October–November 2025 rainfall field supplied to the ABM. Panel (B) shows mean modeled conflict pressure across baseline seeds. Panel (C) reports the mean temporal evolution of conflict pressure, resource inequality, and cooperation. Panel (D) decomposes conflict pressure into power, value-orientation, resource-disparity, and environmental components.

Fig 2 combines the observed environmental forcing, the modeled spatial conflict surface, the mean baseline trajectory, and the decomposition of the conflict index. The baseline conflict surface is spatially concentrated rather than uniform, despite smooth environmental variation over substantial parts of the domain. Across the 20 baseline seeds, inequality rises from 𝒢(0)=0.192 to 𝒢(119)=0.868, while mean cooperation falls from 0.547 to 0.118. Conflict pressure initially rises, peaks during the early transient, and then settles near 0.207 at the final step; mean resources decline from 0.560 to 0.312.

The decomposition in Fig 2D shows that the composition of conflict is itself dynamic. At the final step, the mean contributions per interaction are 0.0342 from power disparity, 0.0657 from value-orientation difference, 0.0207 from resource disparity, and 0.0862 from environmental stress. The corresponding mean seed-level shares are 16.6%, 32.0%, 9.3%, and 42.2%, respectively. Because shares are averaged across seeds separately from component magnitudes, these percentages are not obtained by dividing the mean component values by their summed means. Resource disparity therefore matters, but it is neither the only nor the largest final contributor.

### 3.2. Independent parameter effects in the joint sensitivity experiment

**Fig 3:**
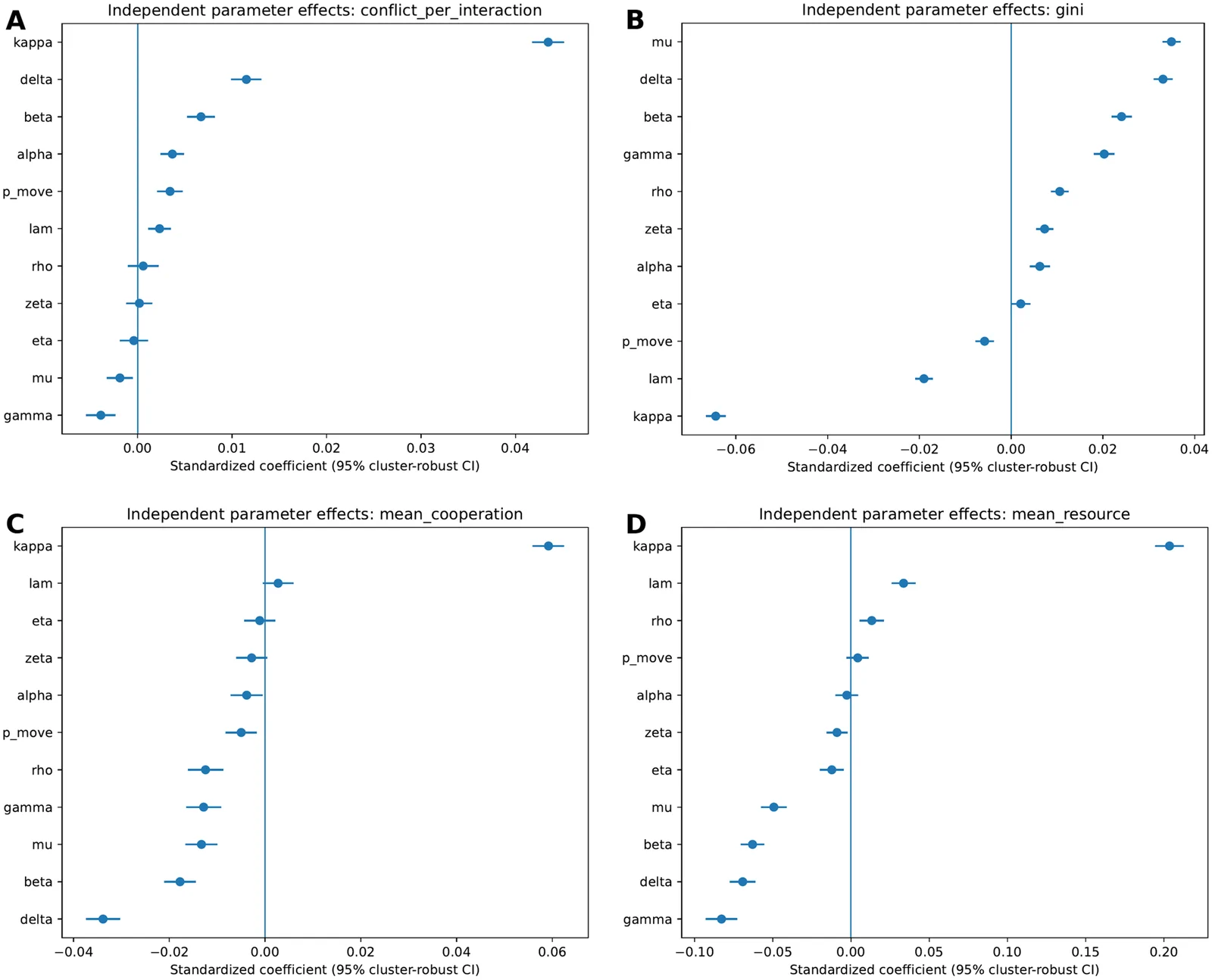
Independent parameter effects from the joint Latin-hypercube sensitivity experiment. Points are standardized multivariable coefficients and horizontal intervals are 95% run-clustered confidence intervals. All parameters are varied jointly, so coefficients represent conditional effects in the simulated ensemble rather than one-at-a-time slopes.

Fig 3 reports the standardized conditional effects from the 640-run Latin-hypercube ensemble. The four outcomes respond to different parameter combinations, which argues against a single-parameter explanation. Institutional support κ has the strongest positive main effect on conflict pressure (0.04346, 95% CI [0.04175,0.04518]), the strongest negative main effect on Gini (−0.06439, 95% CI [−0.06658,−0.06220]), the strongest positive main effect on cooperation (0.05918, 95% CI [0.05587,0.06250]), and the strongest positive main effect on mean resources (0.20354, 95% CI [0.19430,0.21279]). Environmental stress also increases conflict pressure (0.01151, 95% CI [0.00990,0.01312]) and Gini (0.03309, 95% CI [0.03102,0.03515]), while reducing cooperation and resources.

The negative conditional coefficient for γ on conflict pressure (−0.00390, 95% CI [−0.00547,−0.00233]) should not be interpreted as evidence that resource inequality is protective. In the coupled model, γ also changes resource accumulation, cooperation, and future disparity, so the coefficient is conditional on the rest of the joint parameter ensemble. Table 3 gives the principal numerical effects, while Fig 3 displays all 11 swept parameters for all four outcomes.

**Table 3:** Selected standardized main effects from the time-aware multivariable regressions. All listed effects have *p* < 0.001. Full main effects are visualized in Fig 3; complete machine-readable results, including time interactions, are supplied in Supporting Information.

Outcome	Parameter	Coefficient	95% CI low	95% CI high
Conflict/interact.	κ	0.043462	0.041749	0.045175
Conflict/interact.	δ	0.011511	0.009903	0.013119
Conflict/interact.	β	0.006707	0.005243	0.008171
Conflict/interact.	γ	−0.003898	−0.005466	−0.002330
Gini	κ	−0.064389	−0.066579	−0.062199
Gini	μ	0.034926	0.032941	0.036911
Gini	δ	0.033089	0.031023	0.035154
Gini	λ	−0.019016	−0.020950	−0.017082
Cooperation	κ	0.059184	0.055865	0.062503
Cooperation	δ	−0.033844	−0.037403	−0.030286
Cooperation	β	−0.017774	−0.021085	−0.014462
Cooperation	ρ	−0.012443	−0.016147	−0.008738
Mean resource	κ	0.203545	0.194296	0.212793
Mean resource	γ	−0.082741	−0.092890	−0.072592
Mean resource	δ	−0.069203	−0.077441	−0.060964
Mean resource	λ	0.033609	0.025844	0.041373

The final-step PRCC analysis independently supports these multivariable patterns. For conflict pressure, the largest absolute PRCCs are κ=0.570, δ=0.542, λ=0.321, ρ=−0.315, γ=−0.259, and β=0.250. For Gini, the strongest are λ=−0.667, η=−0.584, μ=0.464, ρ=0.426, γ=0.369, and κ=−0.364. Cooperation is dominated by κ=0.871, followed by ρ=−0.673 and δ=−0.653, while mean resources are most strongly related to λ=0.718 and κ=0.640. The complete PRCC matrix is reported in Table B in S1 Appendix.

### 3.3. Parameter effects change over simulation time

**Fig 4:**
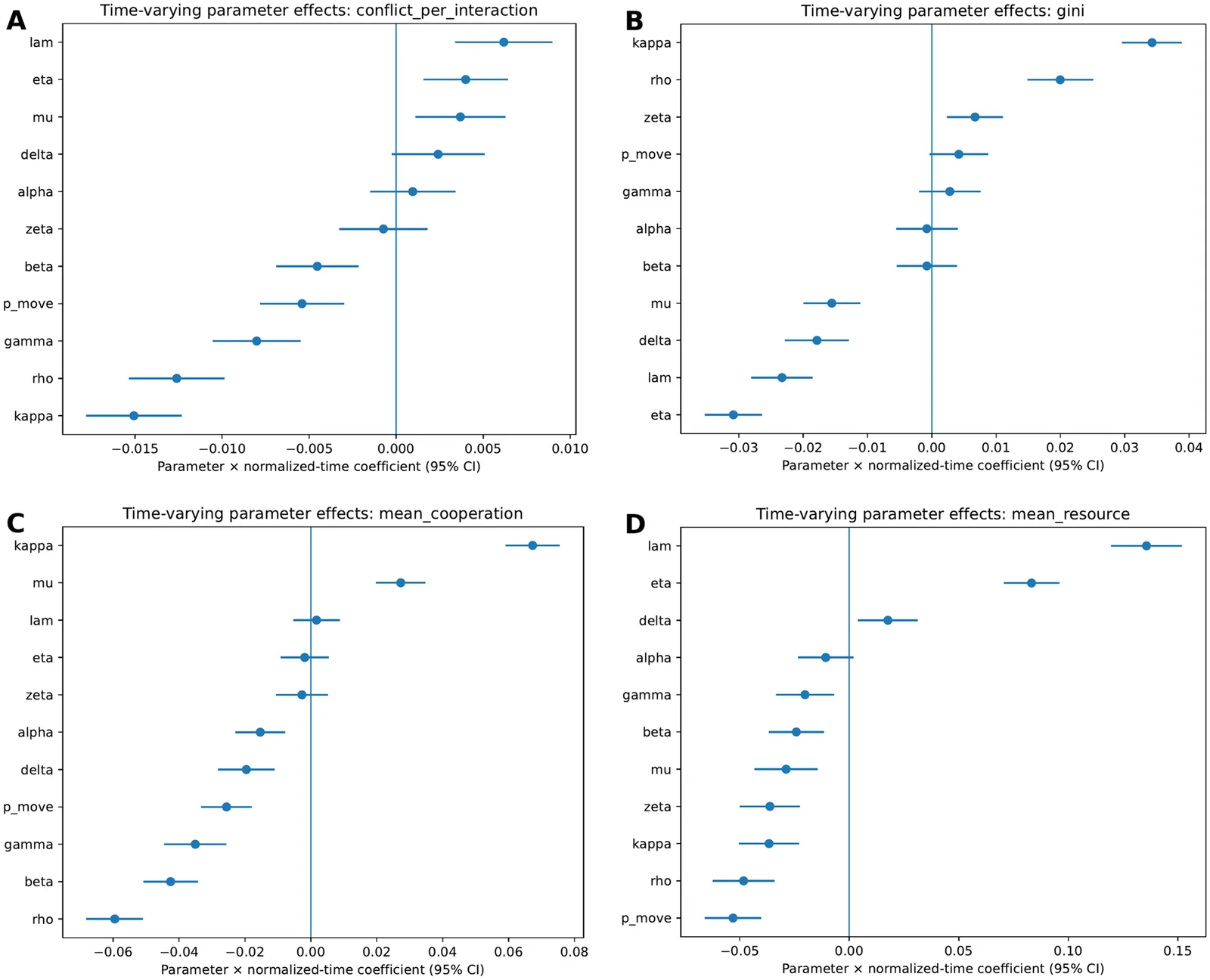
Time-varying parameter effects. Points report parameter-by-normalized-time coefficients and horizontal intervals give 95% run-clustered confidence intervals. The analysis uses complete simulated trajectories rather than only final-step outcomes.

Fig 4 directly addresses the need to include time as an explicit analysis variable. The regression fits are nontrivial (*R*^2^ = 0.537 for conflict pressure, 0.766 for Gini, 0.890 for cooperation, and 0.723 for mean resources), and several parameter-by-time interactions are large relative to their main effects.

Institutional support gives the clearest example. Although the main κ effect on conflict pressure is positive, the κ×t coefficient is negative (−0.01507, 95% CI [−0.01782,−0.01232]), so this additional conflict pressure weakens over the simulation horizon. Conversely, the κ×t effect on cooperation is strongly positive (0.06730, 95% CI [0.05904,0.07556]). The negative main effect of κ on Gini is partly offset over time by a positive κ×t coefficient of 0.03425 (95% CI [0.02959,0.03891]), while its positive resource main effect is partially offset by κ×t=−0.03660 (95% CI [−0.05037,−0.02284]). Resource-production and recovery parameters strengthen over time: λ×t=0.13567 and η×t=0.08318 for mean resources. Thus the ABM cannot be interpreted as a static parameter-to-outcome map.

### 3.4. Institutional-support paradox and structural hard-wiring audit

**Fig 5:**
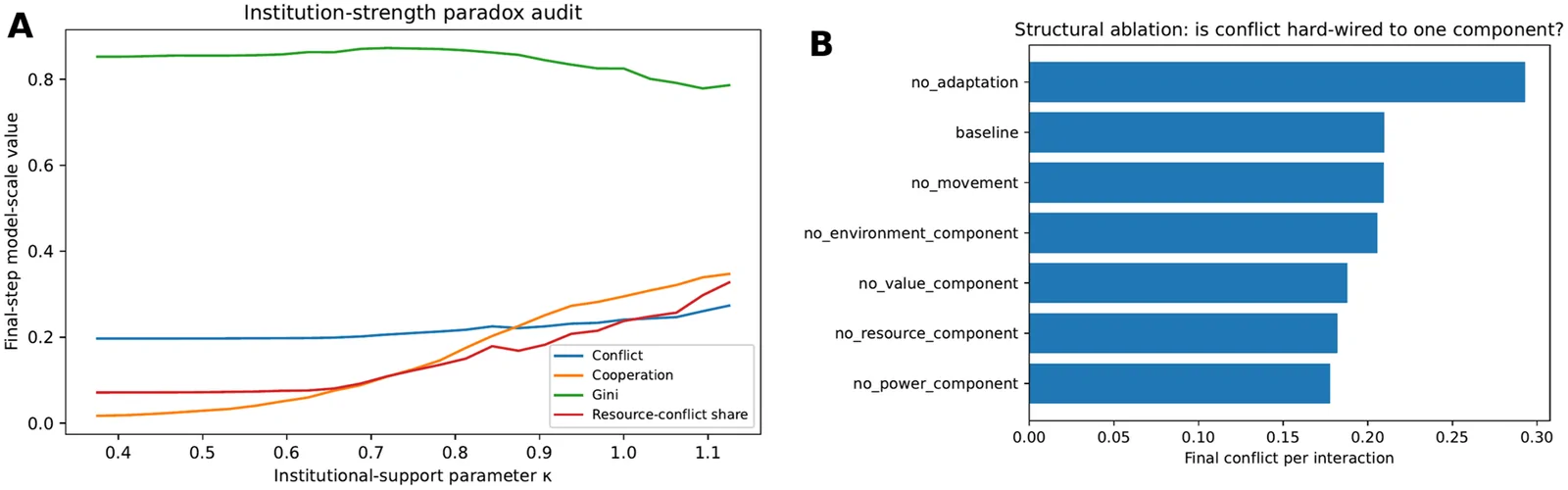
Mechanism audits addressing the counterintuitive institutional scenario and the possibility of mechanically hard-wired conflict. Panel (A) follows conflict, cooperation, inequality, and the resource-related conflict share across institutional support κ. Panel (B) removes each major conflict component and selected adaptive mechanisms separately.

A lower conflict-pressure value under weaker institutional support does not represent a welfare improvement. Fig 5A shows that as κ increases, cooperation rises markedly and inequality falls at the upper part of the tested range, while the resource-related share of conflict pressure also rises. The conflict index therefore increases partly because stronger institutions change the resource state and the relative contribution of resource disparity. At low κ, measured conflict can be lower while cooperation is near collapse and inequality remains high. This resolves the apparent paradox: $S_{ij}$ is an interaction-pressure index, not a social-welfare score.

The structural ablation in Fig 5B directly tests whether conflict is hard-wired to any single component. Mean final conflict pressure across the five ablation seeds is 0.2097 under the baseline. Removing the power component lowers it to 0.1776; removing the resource component lowers it to 0.1821; removing the value component lowers it to 0.1880; and removing the environmental component leaves 0.2058. None of these removals eliminates conflict. In contrast, removing behavioral adaptation raises final conflict to 0.2930, approximately 39.7% above baseline. Removing movement changes conflict only slightly (0.2094). These results show that resource disparity is consequential but not uniquely determinative and that endogenous adaptation materially changes the outcome.

### 3.5. Nonlinear response regions and robustness

The dense one-at-a-time sweeps reveal nonlinear response regions rather than a single universal tipping point. The steepest local cooperation response to institutional support occurs near κ=0.8125, while the steepest Gini and conflict responses occur near κ=1.03125 and κ=1.09375, respectively. For environmental stress, the steepest conflict response occurs near δ=0.1875 and the steepest cooperation response near δ=0.1000. These locations are diagnostics of local slope, not formal bifurcation thresholds. The complete curves are shown in Fig A in S1 Appendix, and all candidate transition locations are listed in Table A in S1 Appendix.

Resolution checks in Table D in S1 Appendix show that conflict pressure remains of the same order across the four tested grid/agent configurations (mean final values 0.1885–0.2188). Distributional and cooperation outcomes are more resolution-sensitive, particularly when agent density changes. This is reported as a limitation rather than hidden: the qualitative conflict mechanism is robust to the tested numerical resolutions, whereas Gini and cooperation require caution when comparing runs with different spatial/agent densities.

## 4. Discussion

The analysis provides three complementary lines of evidence. First, sensitivity is no longer inferred mainly from a small set of hand-designed scenarios. The 128-design Latin-hypercube experiment jointly varies all continuous parameters, and both cluster-robust regression and final-step PRCC identify parameter-specific effects. Second, time is included explicitly. Parameter-by-time interactions show that several mechanisms change sign or magnitude over the trajectory, so final-step comparisons alone are inadequate. Third, structural ablation separates a model component from the feedback it induces. Resource disparity contributes to conflict pressure by construction, but the ablation results show that the observed aggregate pattern is not reducible to that term alone.

The institutional-support result is particularly important for interpretation. Stronger institutions increase cooperative gain by construction, but their system-wide effects are not equivalent to simply “lower conflict.” Higher κ improves cooperation and resources and reduces inequality over much of the tested range, while also altering the resource component of the conflict index. The apparently paradoxical low-conflict state under weak institutions is therefore a degraded state with weak cooperation, not evidence that institutional weakness is beneficial. This result reinforces the need to evaluate conflict pressure, inequality, cooperation, and resources jointly.

The time-aware results also clarify the role of environmental parameters. Environmental stress has a clear positive main effect on conflict and inequality and a negative effect on cooperation and resources. At the same time, recovery and terrain contribution become more important for resources as the system evolves. This is consistent with a feedback interpretation in which environmental conditions affect both immediate interaction pressure and the long-run resource state.

The paper does not treat the candidate transition locations as universal thresholds. They identify where the simulated response curve is steepest within the declared parameter interval. Their practical value is to indicate parameter regions in which small changes produce comparatively large model responses and therefore deserve closer scrutiny in calibration or policy stress testing.

### 4.1. Validation scope and limitations

Several limitations remain. First, the Uganda run is spatially explicit with observed WorldPop population and CHIRPS rainfall, but no observed georeferenced conflict-event surface is matched to the same October–November 2025 window. The modeled conflict surface in Fig 2B is therefore a spatial model diagnostic rather than an observed-event validation. Second, rainfall is used as a terrain-quality proxy; future applications should use NDVI, land condition, water access, or other location-specific ecological indicators when available. Third, the initialization of power from population exposure is a transparent proxy rather than a direct measure of institutional or political power. Fourth, the resolution audit shows that inequality and cooperation are more sensitive than conflict pressure to agent density and grid scale. Fifth, the model omits explicit ethnic identity, bargaining institutions, multi-level authority, displacement, and case-specific political shocks. The appropriate interpretation is therefore mechanism exploration and stress testing, not event forecasting.

## 5. Conclusion and future directions

This study develops a spatial agent-based framework in which conflict pressure, resource inequality, cooperation, movement, and environmental quality co-evolve. The analysis goes beyond scenario illustration by jointly varying the model parameters, estimating independent effects, including time explicitly, identifying steep-response regions, testing structural ablations, and checking numerical resolution.

The central result is that conflict pressure cannot be interpreted in isolation. The baseline trajectory shows rising inequality and collapsing cooperation even while conflict pressure stabilizes. Global sensitivity shows that institutional support can simultaneously increase modeled interaction pressure, reduce inequality, and increase cooperation and resources. Structural ablation shows that resource disparity is important but does not mechanically determine the conflict outcome. The apparent weaker-institutions paradox is therefore an interpretive property of a multi-output feedback system rather than evidence that weak institutions improve welfare.

The framework is best used to compare mechanisms, expose trade-offs, and identify sensitive regions of parameter space. Operational use would require case-specific institutional indicators, stronger ecological proxies, and observed subnational conflict outcomes matched to the same spatial and temporal window. With those additions, the model could support conflict-sensitive resource planning while retaining the transparency needed for data-constrained settings.

## Supporting information

S1 Appendix Sensitivity, transition, and robustness diagnostics. Supplementary one-at-a-time response curves, candidate steep-response locations, complete partial-rank sensitivity results, structural-ablation summaries, and grid/agent-count robustness checks.
(PDF)

S1 Data Primary machine-readable analysis tables. Contains baseline_mean_trajectory.csv, baseline_seed_trajectories.csv, parameter_lhs_design.csv, parameter_ranges_and_justification.csv, parameter_time_sweep.csv, time_panel_regression.csv, partial_rank_correlations.csv, one_at_a_time_sweeps.csv, candidate_transition_points.csv, structural_ablation.csv, and resolution_robustness.csv.
(ZIP)

S1 File Supporting-information package guide. README describing the organization, analysis provenance, and distinction between primary Uganda-focused outputs and contextual legacy outputs.
(TXT)

S2 Data Contextual country-level summary tables. Country-level GED/CHIRPS summary and exploratory alignment tables retained for transparency; these are not used as the primary evidence for the revised Uganda analysis.
(ZIP)

S2 File Contextual country-level diagnostic figures. Administrative-unit descriptive alignment plots and country-level trajectories for Egypt, Ethiopia, Ghana, Kenya, Madagascar, Nigeria, Somalia, Tanzania, and Uganda, retained for transparency only and not presented as raster-level validation.
(PDF)

S1 Code Reproducibility code for the spatial ABM and robustness analyses. Python source code for the spatial ABM, global sensitivity analysis, time-aware regression, one-at-a-time diagnostics, ablation analysis, and resolution checks.
(ZIP)
