# Supplementary material for: Modeling resource conflict and inequality in fragile systems: A spatial agent-based framework with a Uganda case study
Source: PLoS One. 2026 Sep 16;21(9):e0357283. doi: 10.1371/journal.pone.0357283 (PMC13581203; doi:10.1371/journal.pone.0357283)
Supplement: Supplementary file 1 [file pone.0357283.s001.pdf]

S1 Appendix. Sensitivity, Transition, and Robustness Diagnostics

This appendix contains supporting analyses that are useful for reproducibility but too detailed for the main text. It reports the complete one-at-a-time response curves, partial-rank sensitivity matrix, candidate steep-response locations, structural-ablation summaries, and grid/agent-count robustness checks. Fig A and Tables A–D are each cited and discussed below.

0.1 One-at-a-time response curves and candidate transition locations

Fig A reports the dense one-at-a-time sweeps for δ, γ, κ, ρ, and ζ. Each curve averages five stochastic seeds at each of 25 parameter values. The curves confirm that the outputs are nonlinear and that a single parameter can improve one outcome while worsening another. In particular, increasing κ raises cooperation and mean resources strongly over the upper part of its tested interval while eventually reducing Gini, whereas the conflict-pressure response rises nonlinearly. The corresponding steepest local-response locations are summarized numerically in Table A. These values are diagnostics, not formal bifurcation thresholds.

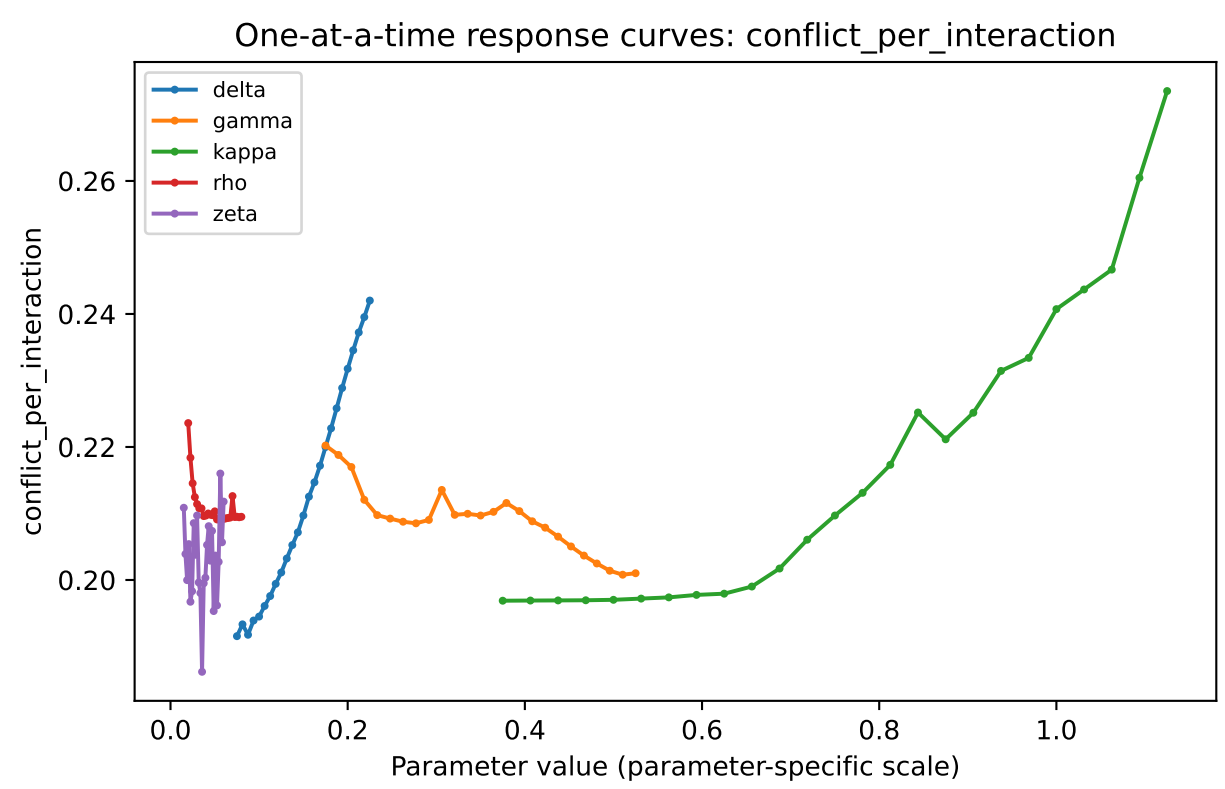

(a) Conflict pressure.

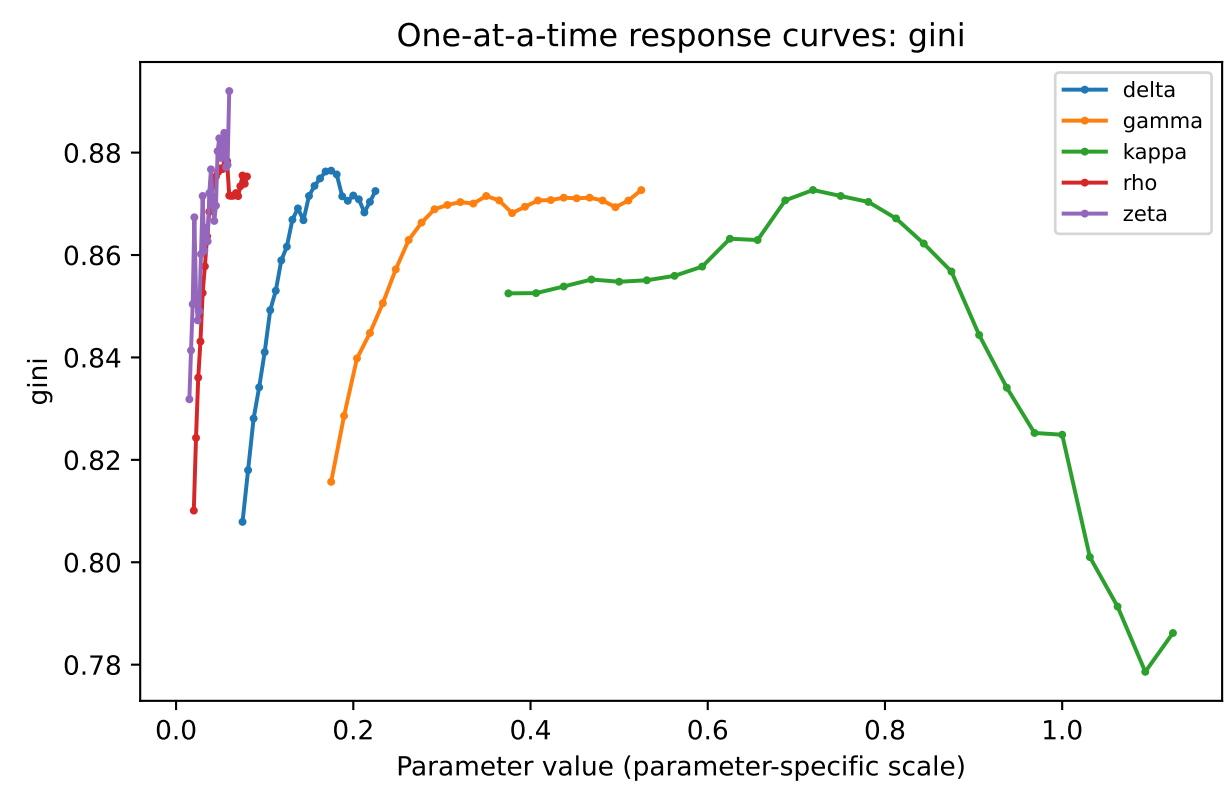

(b) Resource inequality.

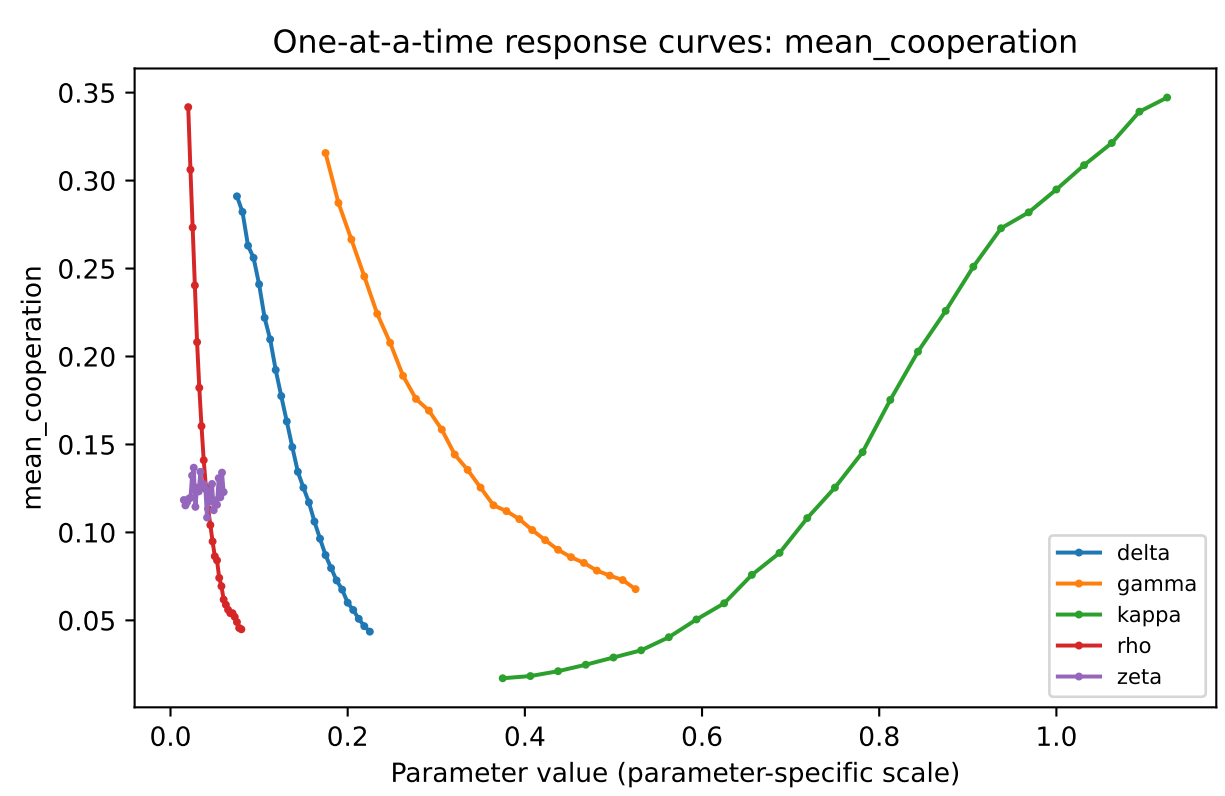

(c) Mean cooperation.

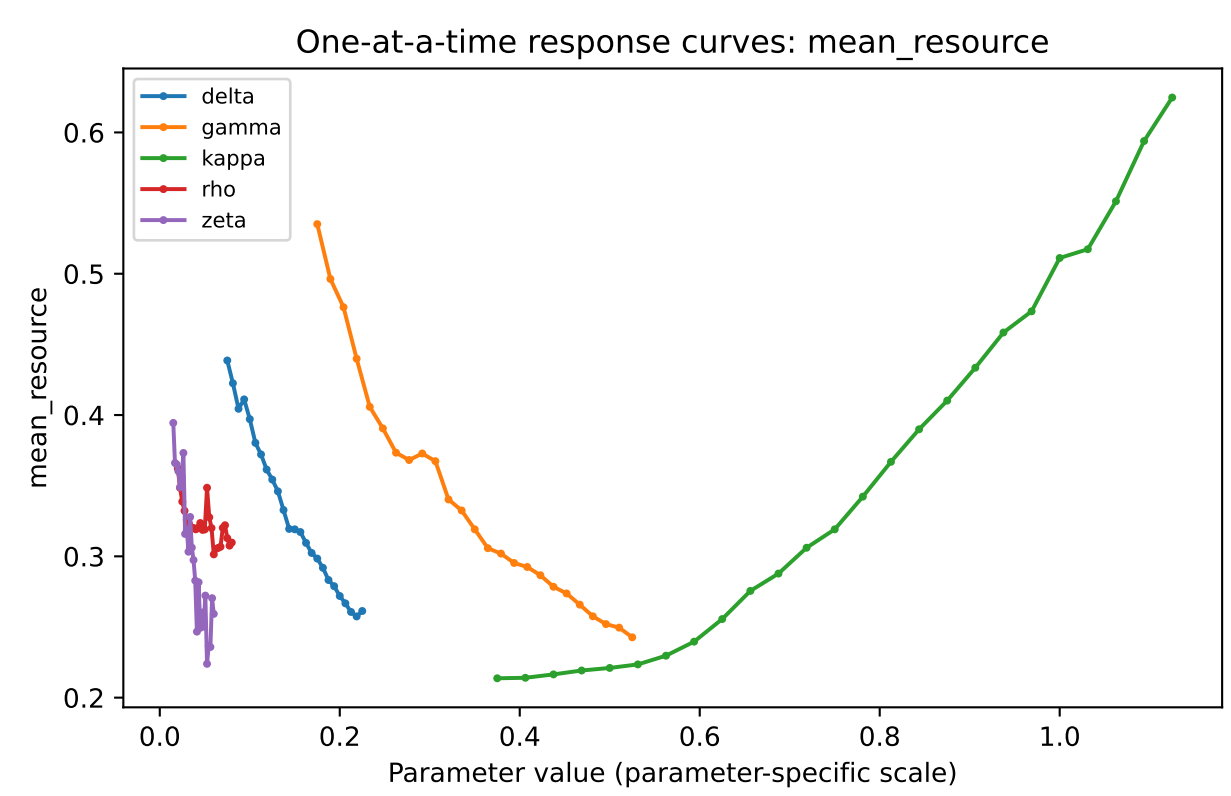

(d) Mean resources.

Fig A: One-at-a-time response curves for the five parameters selected for transition diagnostics. Curves are final-step means over five seeds at each of 25 parameter values. Different parameters have different numerical scales, so the horizontal axis should be interpreted parameter by parameter.

Table A shows that the steepest response depends on both parameter and outcome. The dispersion of these locations is evidence against a single universal "tipping point" in the model. For example, the strongest local cooperation response to κ occurs near 0.8125, whereas the steepest local conflict response occurs near 1.09375.

Table A: Candidate steep-response locations from the one-at-a-time sweeps. Each entry is the sampled parameter value with the largest absolute finite-difference slope for that outcome. These are not formal bifurcation thresholds.

Parameter	Conflict/interact.	Gini	Cooperation	Mean resource
δ	0.187500	0.075000	0.100000	0.081250
γ	0.218750	0.175000	0.175000	0.175000
κ	1.093750	1.031250	0.812500	1.062500
ρ	0.020000	0.020000	0.020000	0.050000
ζ	0.054375	0.060000	0.060000	0.015000

0.2 Complete partial-rank sensitivity matrix

Table B reports the complete PRCC matrix from the 640 final-step runs. The matrix complements the standardized regression coefficients in the main text by using rank residualization. With five exceptions noted below the table, the reported PRCCs are statistically different from zero at the 5% level. The dominant patterns are the strong positive effects of κ on cooperation and resources, the strong negative effect of λ on Gini, and the strong negative effects of ρ and δ on cooperation.

Table B: Partial rank correlation coefficients (PRCCs) from the 640-run Latin-hypercube ensemble at the final simulation step.

Parameter	Conflict/interact.	Gini	Cooperation	Mean resource
α	0.177	0.100	-0.196	-0.094
β	0.250	0.348	-0.591	-0.357
γ	-0.259	0.369	-0.513	-0.366
δ	0.542	0.252	-0.653	-0.249
κ	0.570	-0.364	0.871	0.640
λ	0.321	-0.667	0.153	0.718
μ	-0.034	0.464	0.086	-0.410
η	0.167	-0.584	0.019	0.436
ζ	-0.043	0.302	-0.150	-0.267
ρ	-0.315	0.426	-0.673	-0.153
p_m	-0.027	-0.047	-0.546	-0.316

The nonsignificant PRCC entries in Table B are μ–conflict ($p = 0.397$), ζ–conflict ($p = 0.275$), p_m–conflict ($p = 0.494$), p_m–Gini ($p = 0.237$), and η–cooperation ($p = 0.633$). All other entries have $p < 0.05$ in the final-step rank-residualization analysis.

0.3 Structural-ablation numerical summary

The mechanism-audit figure in the main text is supported numerically by Table C, which averages the five ablation seeds. The table confirms that removing any single conflict-index component leaves substantial residual conflict. It also shows that some structural changes have large effects on the other state variables, reinforcing the need to avoid interpreting conflict pressure as a welfare measure.

Table C also clarifies why ablation outcomes should be interpreted structurally rather than as policy scenarios. For example, removing the resource-conflict term permits resources to accumulate to a very different model-scale regime. The relevant conclusion is not that the ablated system is realistic, but that conflict does not mathematically collapse when the resource-disparity term is removed.

Table C: Mean final-step outcomes across five seeds for each structural ablation.

Ablation	Conflict/interact.	Gini	Cooperation	Mean resource	Resource share
Baseline	0.2097	0.8715	0.1255	0.3191	0.1227
No power component	0.1776	0.8609	0.1781	0.3490	0.1641
No value component	0.1880	0.7516	0.3629	0.5274	0.3578
No resource component	0.1821	0.5693	0.6071	10.4356	0.0000
No environment component	0.2058	0.7280	0.3840	0.6027	0.5153
No adaptation	0.2930	0.7016	0.5458	0.5379	0.3731
No movement	0.2094	0.8775	0.5528	6.9984	0.1765

0.4 Grid and agent-count robustness

Table D reports the five-seed mean outcomes for the four numerical configurations used in the robustness audit. Conflict pressure remains within a relatively narrow range, whereas Gini and cooperation are more sensitive to agent density and grid size. This pattern supports the qualitative conflict mechanism but cautions against comparing distributional quantities across different numerical resolutions without re-calibration.

Table D: Resolution and agent-count robustness. Values are final-step means across five seeds for each configuration.

Grid	Agents	Conflict/interact.	Gini	Cooperation	Mean resource
40	125	0.2188	0.8999	0.0124	0.1766
60	250	0.2004	0.9143	0.0119	0.1253
80	250	0.2097	0.8715	0.1255	0.3191
80	500	0.1885	0.9446	0.0143	0.1183

As shown in Table D, the range of mean final conflict is 0.1885–0.2188 across tested configurations. The larger variation in cooperation and Gini is explicitly acknowledged in the main-text limitations.

0.5 Appendix summary

Fig A and Tables A, B, C, and D provide the supporting evidence for the sensitivity, nonlinear-response, structural, and numerical-robustness claims. The earlier country-level administrative-unit alignment plots and country-by-country trajectory graphics are not reproduced in this Appendix; they are supplied separately as S2 File for transparency and are not used in the revised primary validation claims.
