# Supplementary material for: Modeling resource conflict and inequality in fragile systems: A spatial agent-based framework with a Uganda case study
Source: PLoS One. 2026 Sep 16;21(9):e0357283. doi: 10.1371/journal.pone.0357283 (PMC13581203; doi:10.1371/journal.pone.0357283)

S2 File. Contextual country-level diagnostic figures

These 39 diagnostic figures are retained from the earlier exploratory analysis for transparency. They are not used as the primary evidence for the revised Uganda-focused analysis and are not presented as raster-level predictive validation.

Contents: three administrative-unit descriptive alignment plots plus four country-level model trajectories for each of Egypt, Ethiopia, Ghana, Kenya, Madagascar, Nigeria, Somalia, Tanzania, and Uganda.

1. Administrative-unit descriptive alignment - Rainfall vs GED events per million

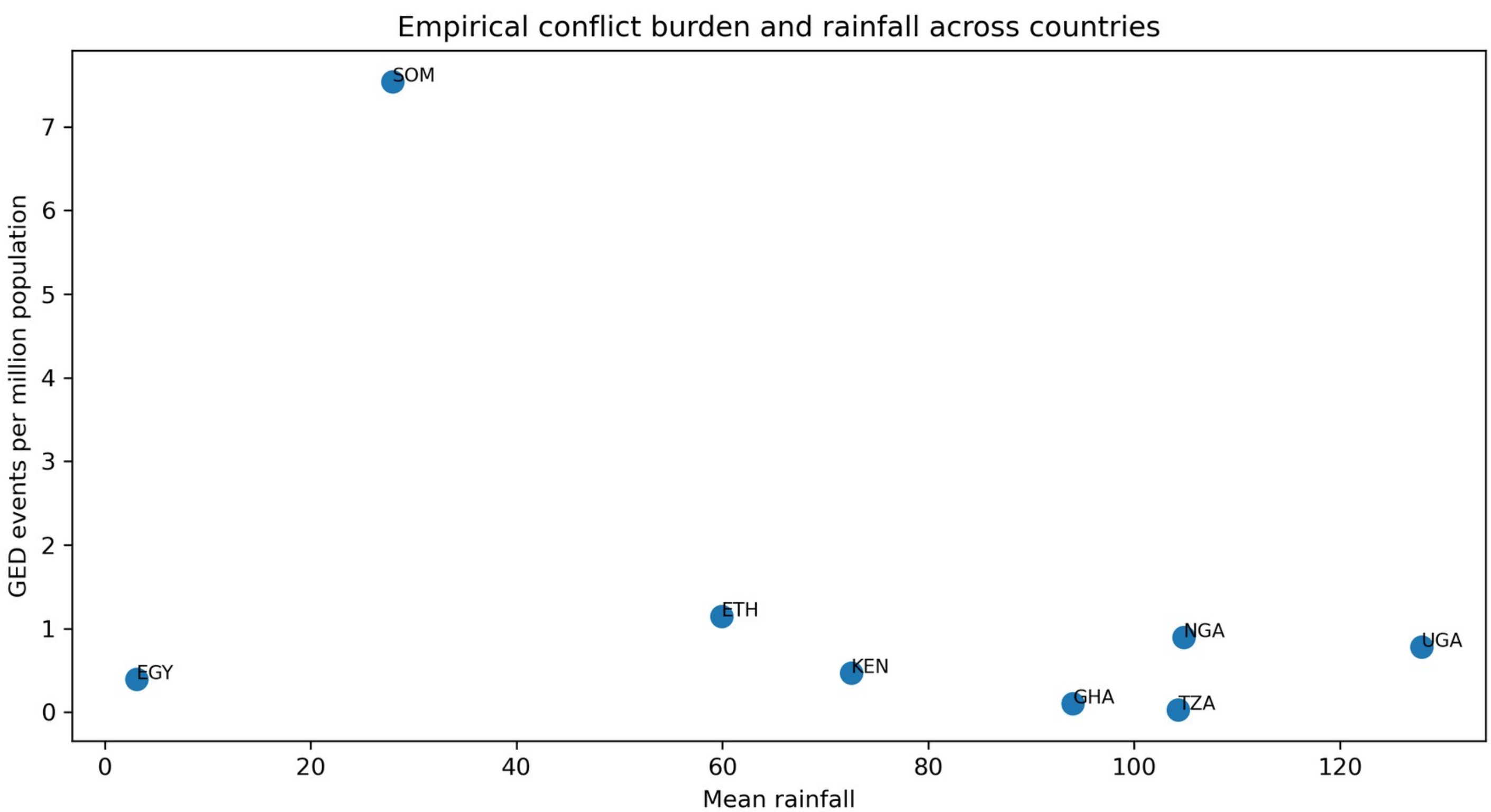

2. Administrative-unit descriptive alignment - GED events per million vs modeled conflict

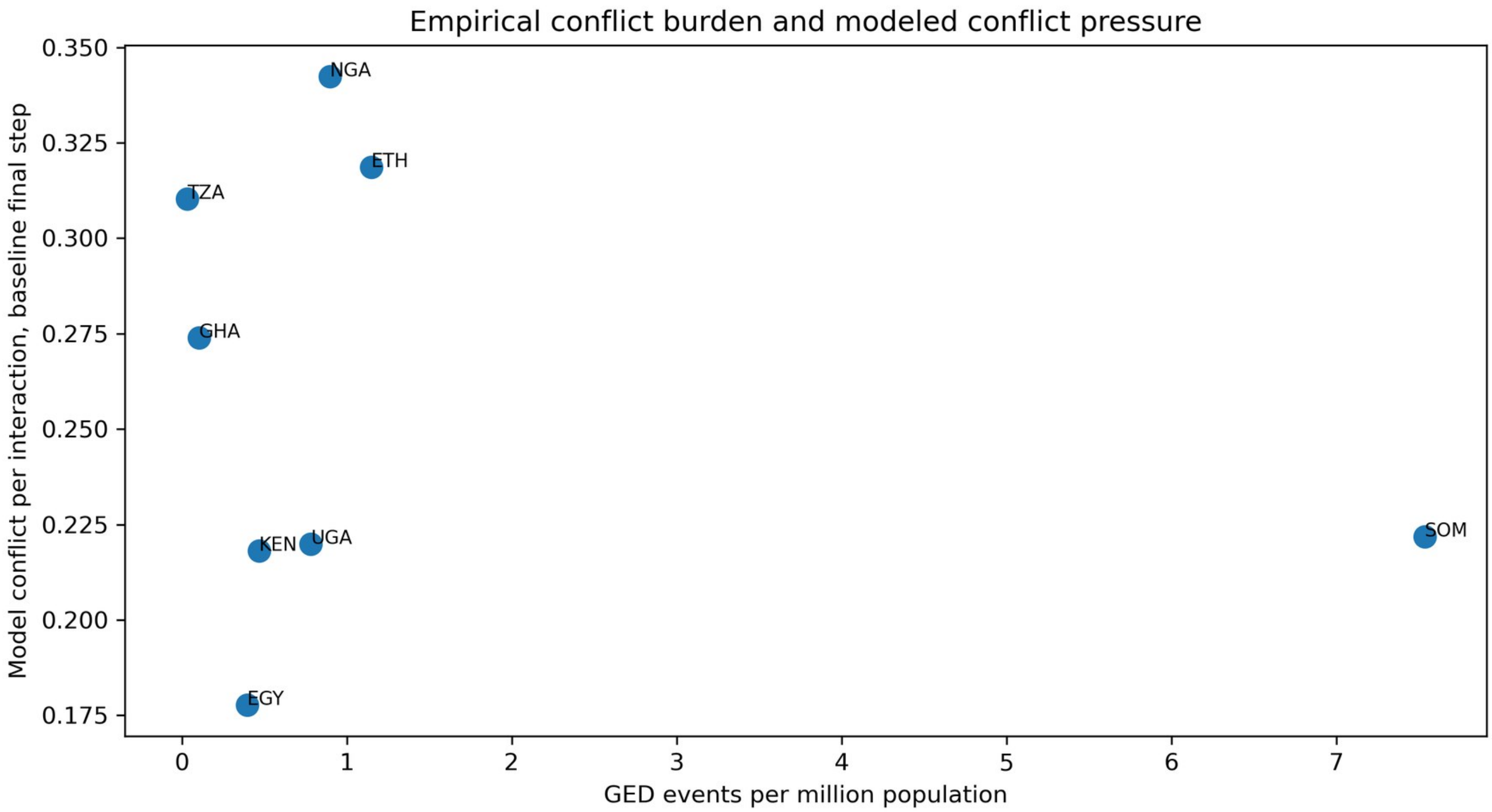

3. Administrative-unit descriptive alignment - GED events per million vs modeled Gini

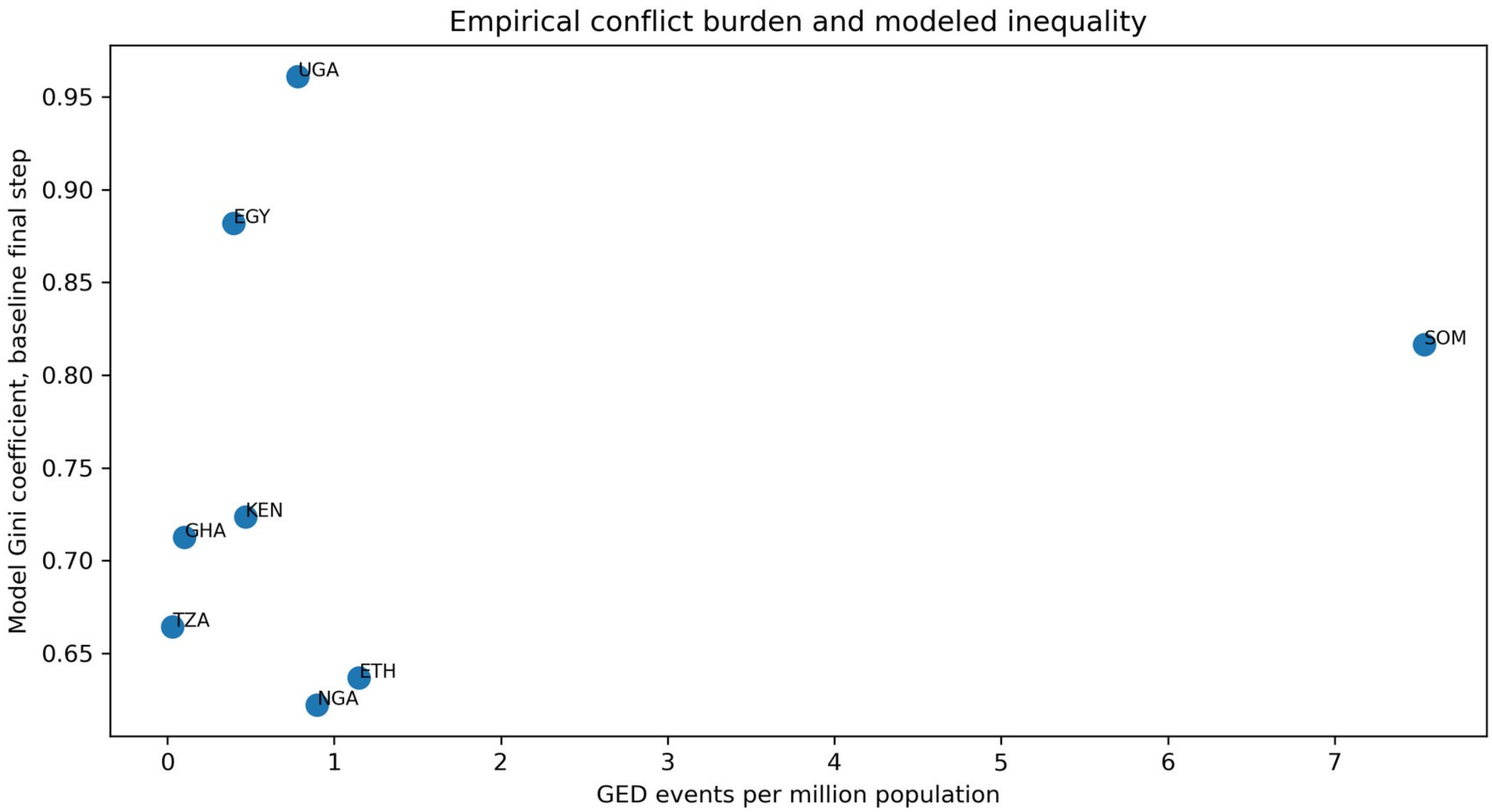

4. Egypt - Conflict-pressure trajectory

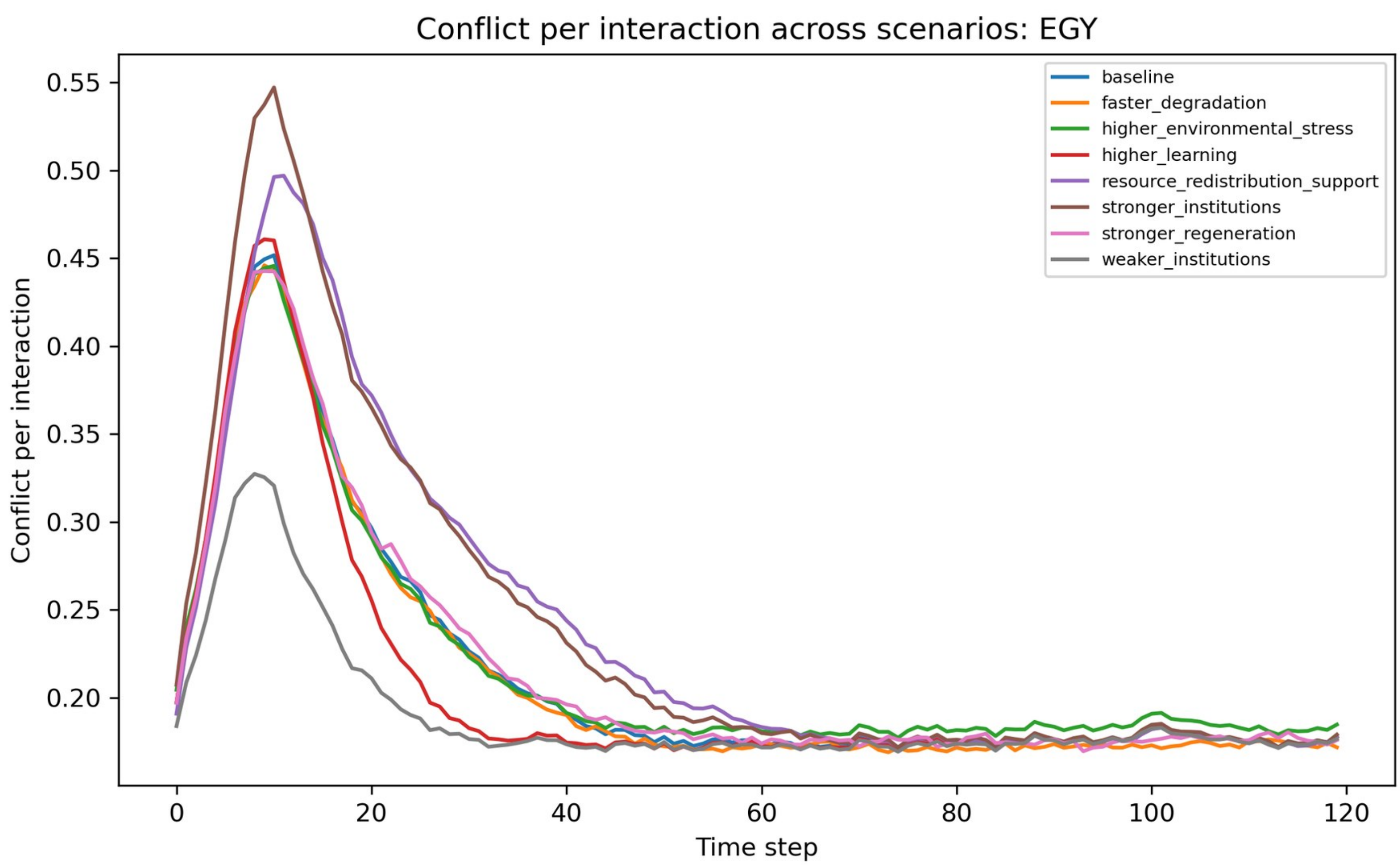

5. Egypt - Gini trajectory

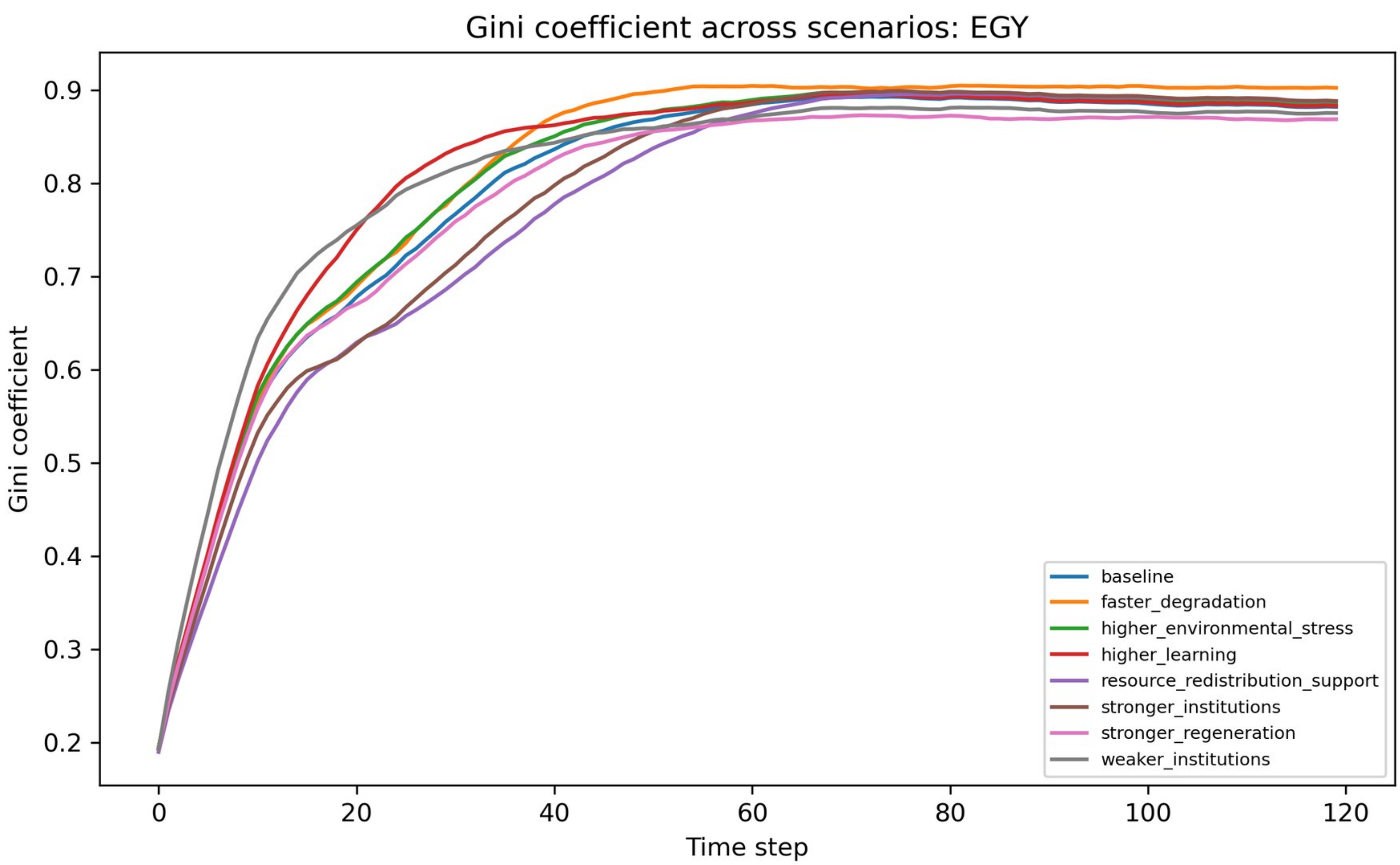

6. Egypt - Cooperation trajectory

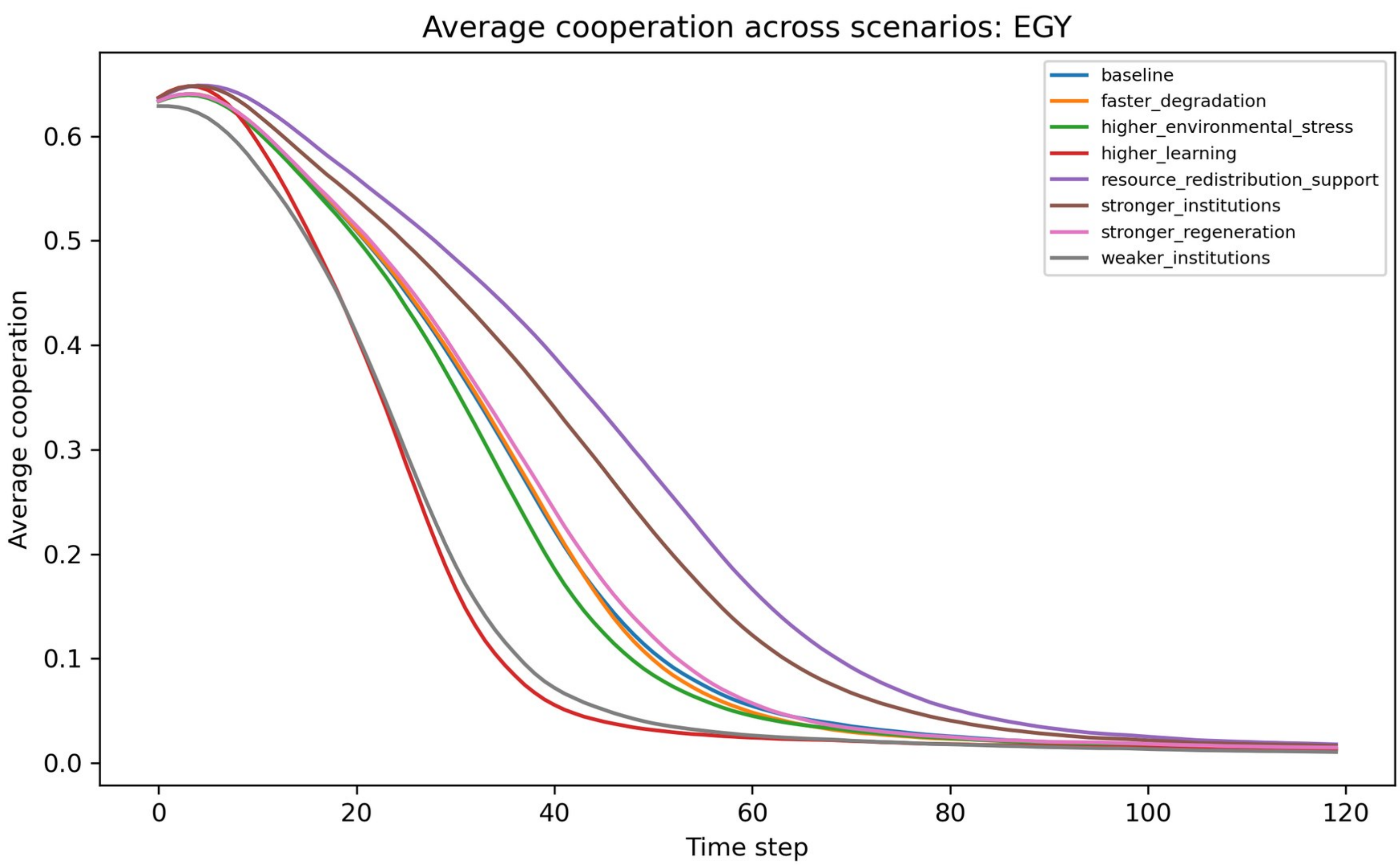

7. Egypt - Mean-resource trajectory

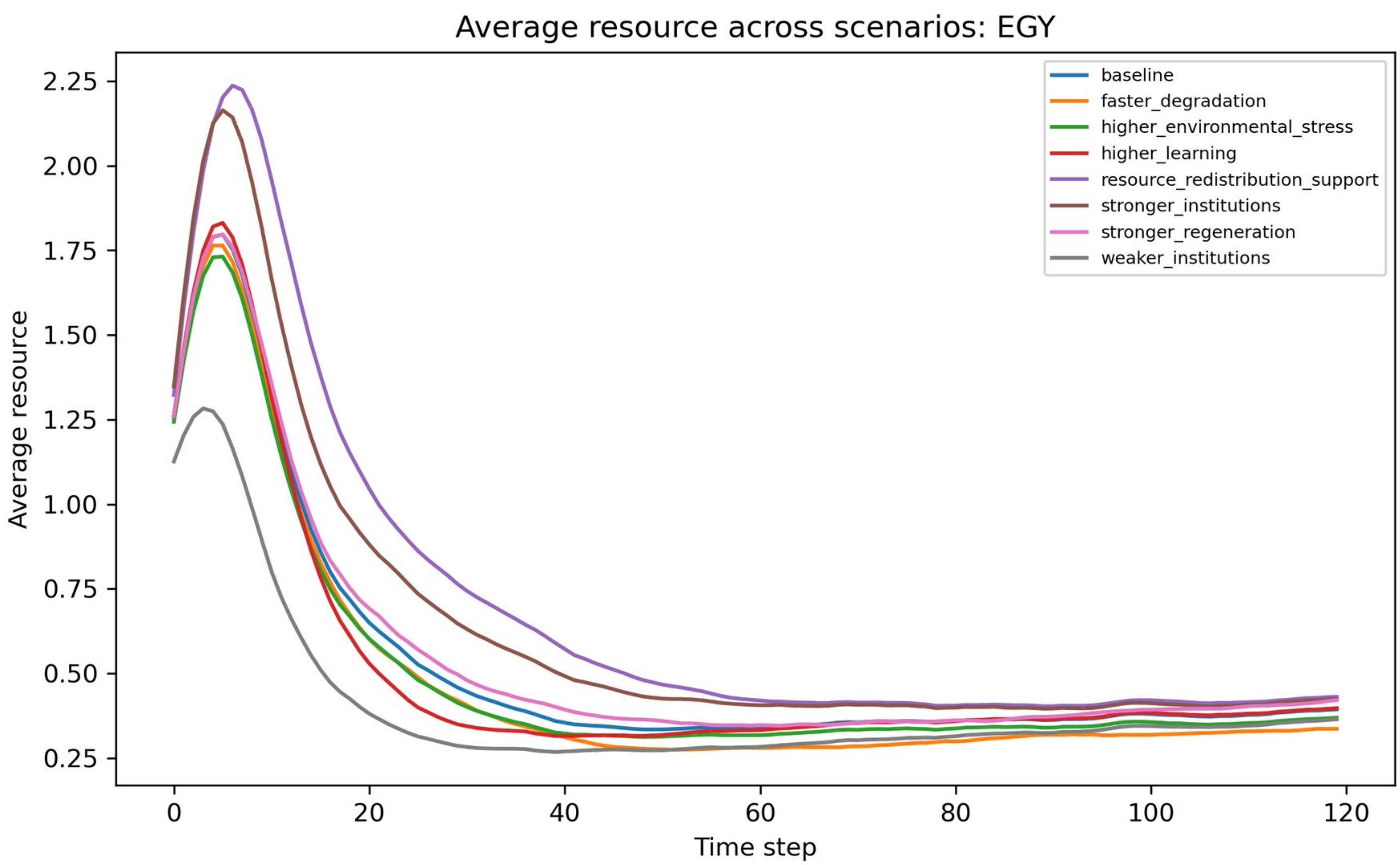

8. Ethiopia - Conflict-pressure trajectory

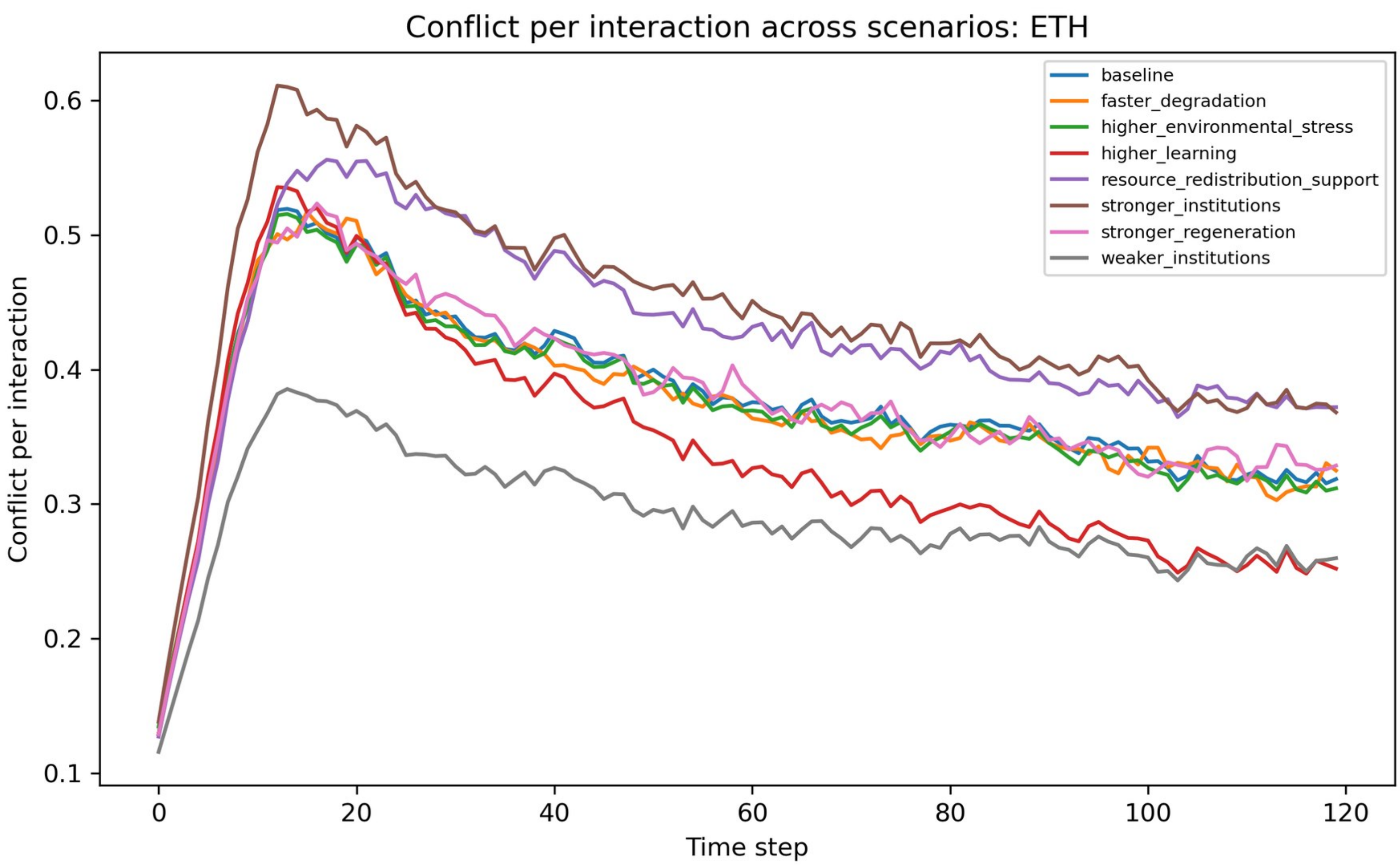

9. Ethiopia - Gini trajectory

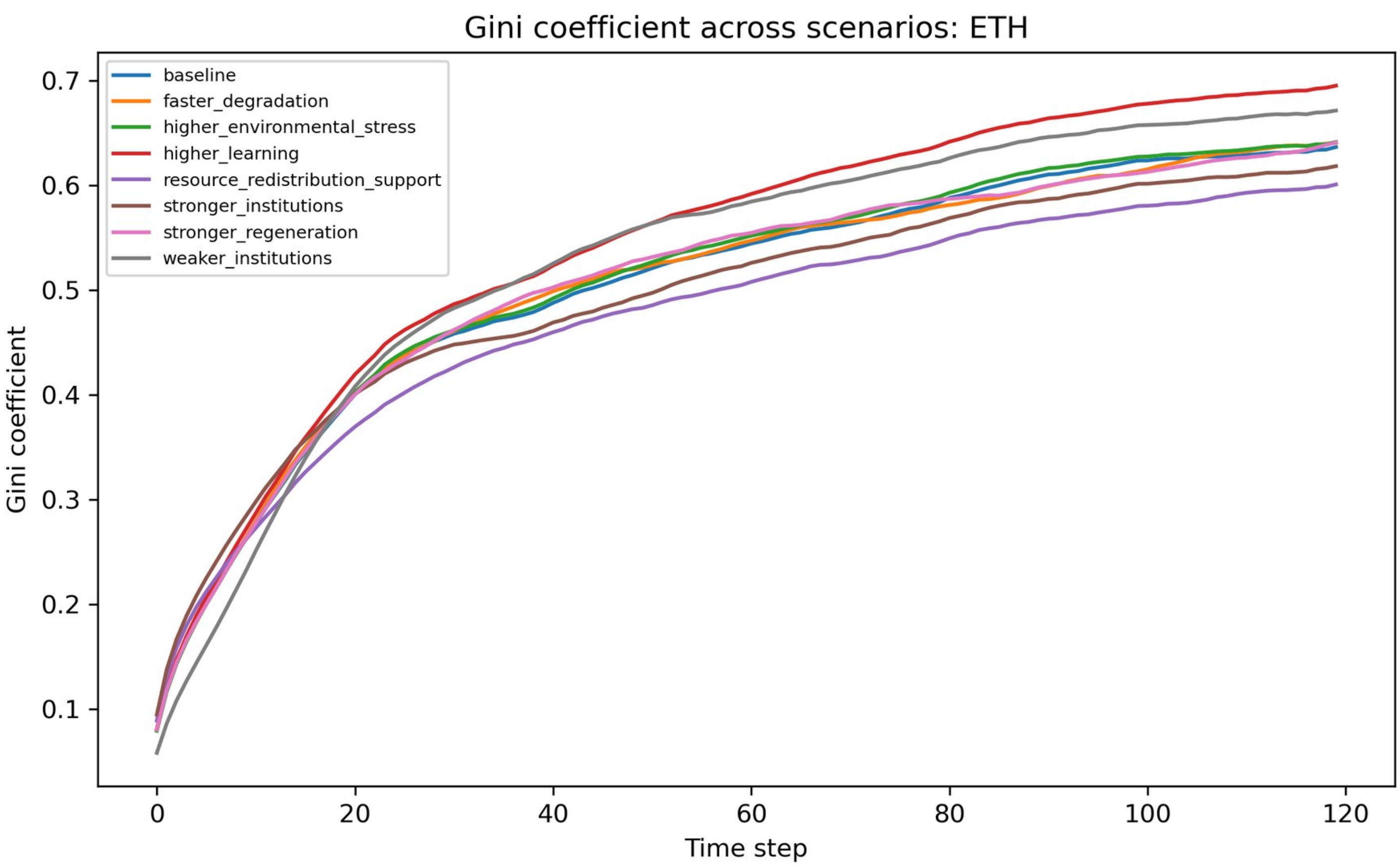

10. Ethiopia - Cooperation trajectory

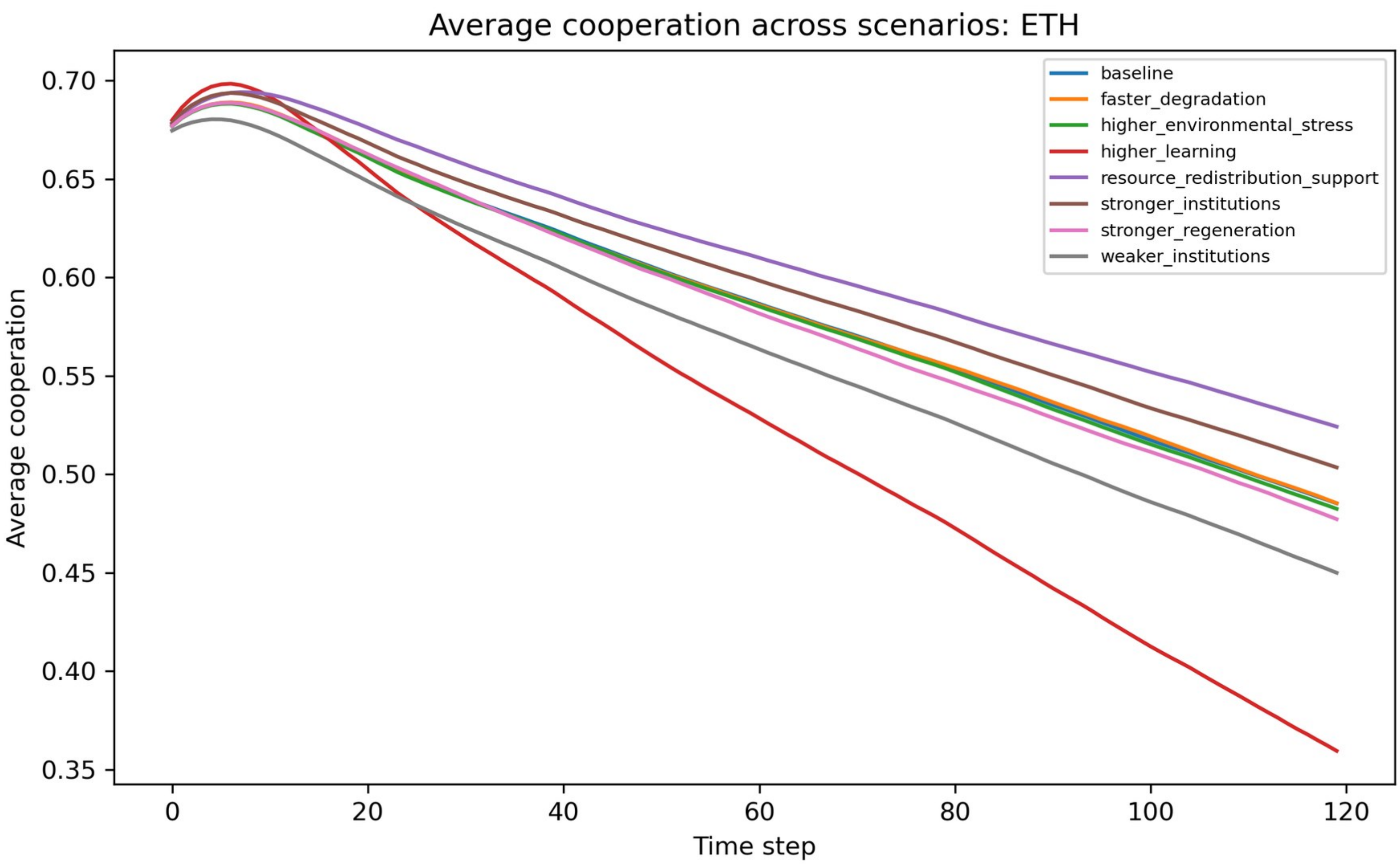

11. Ethiopia - Mean-resource trajectory

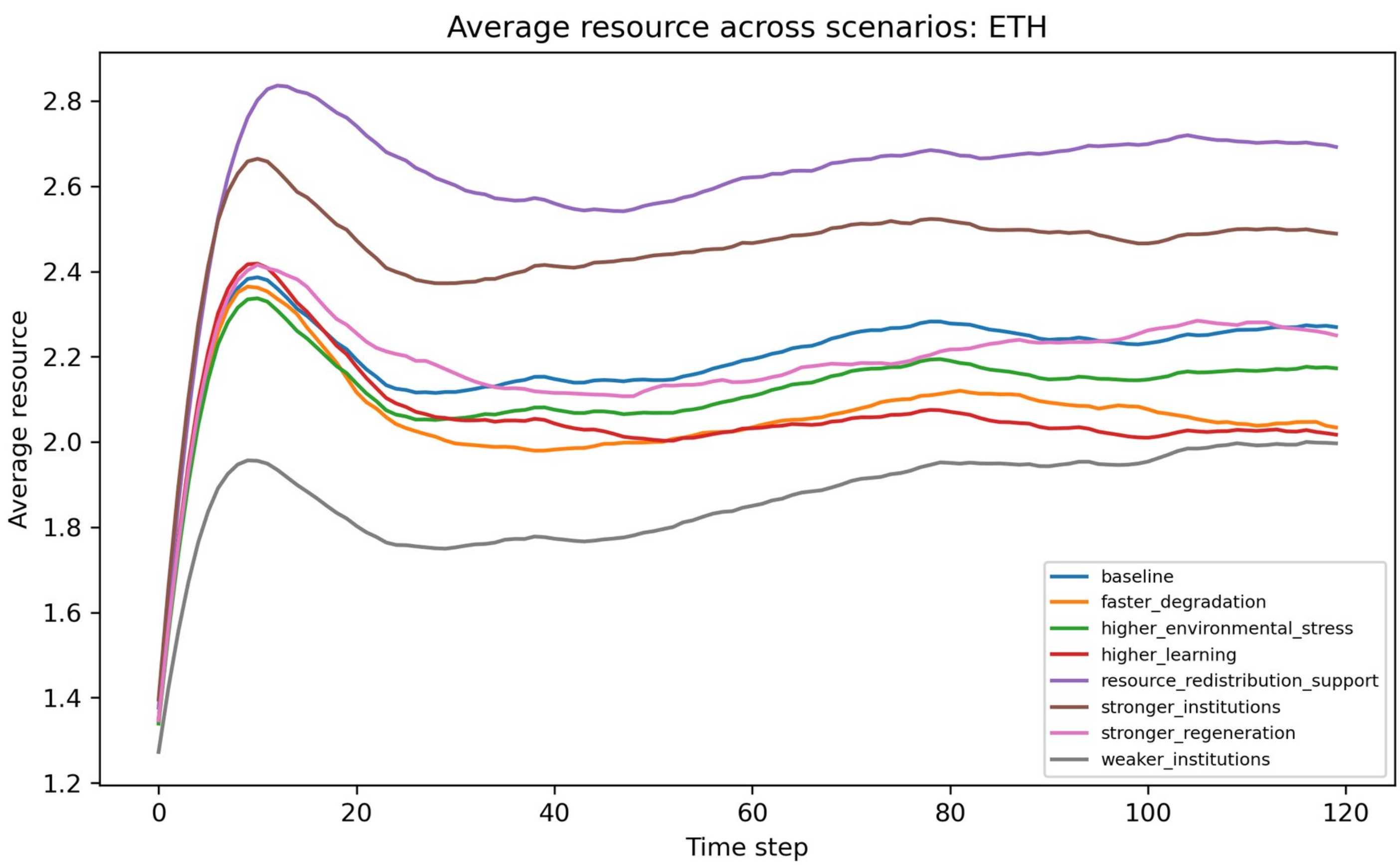

12. Ghana - Conflict-pressure trajectory

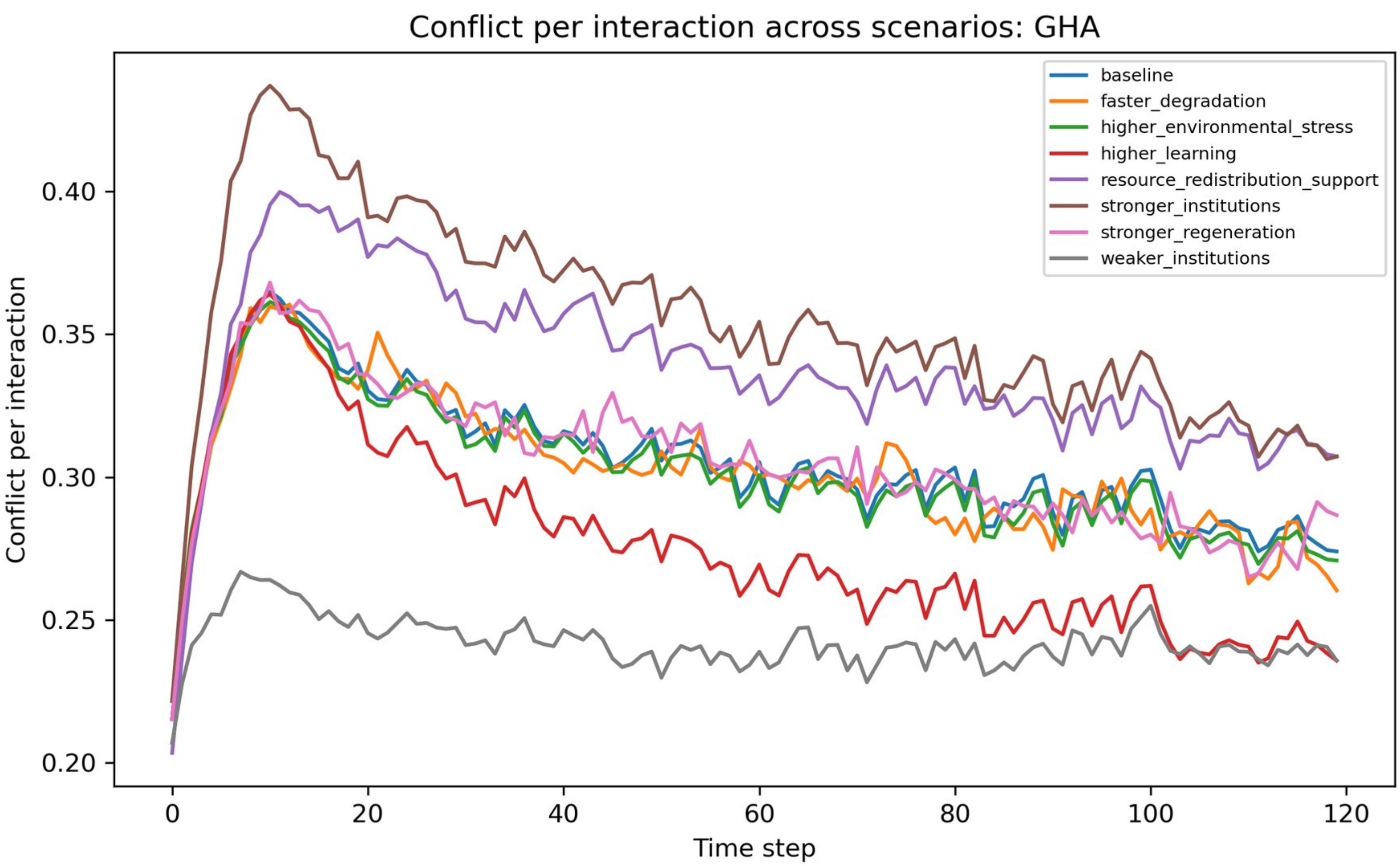

13. Ghana - Gini trajectory

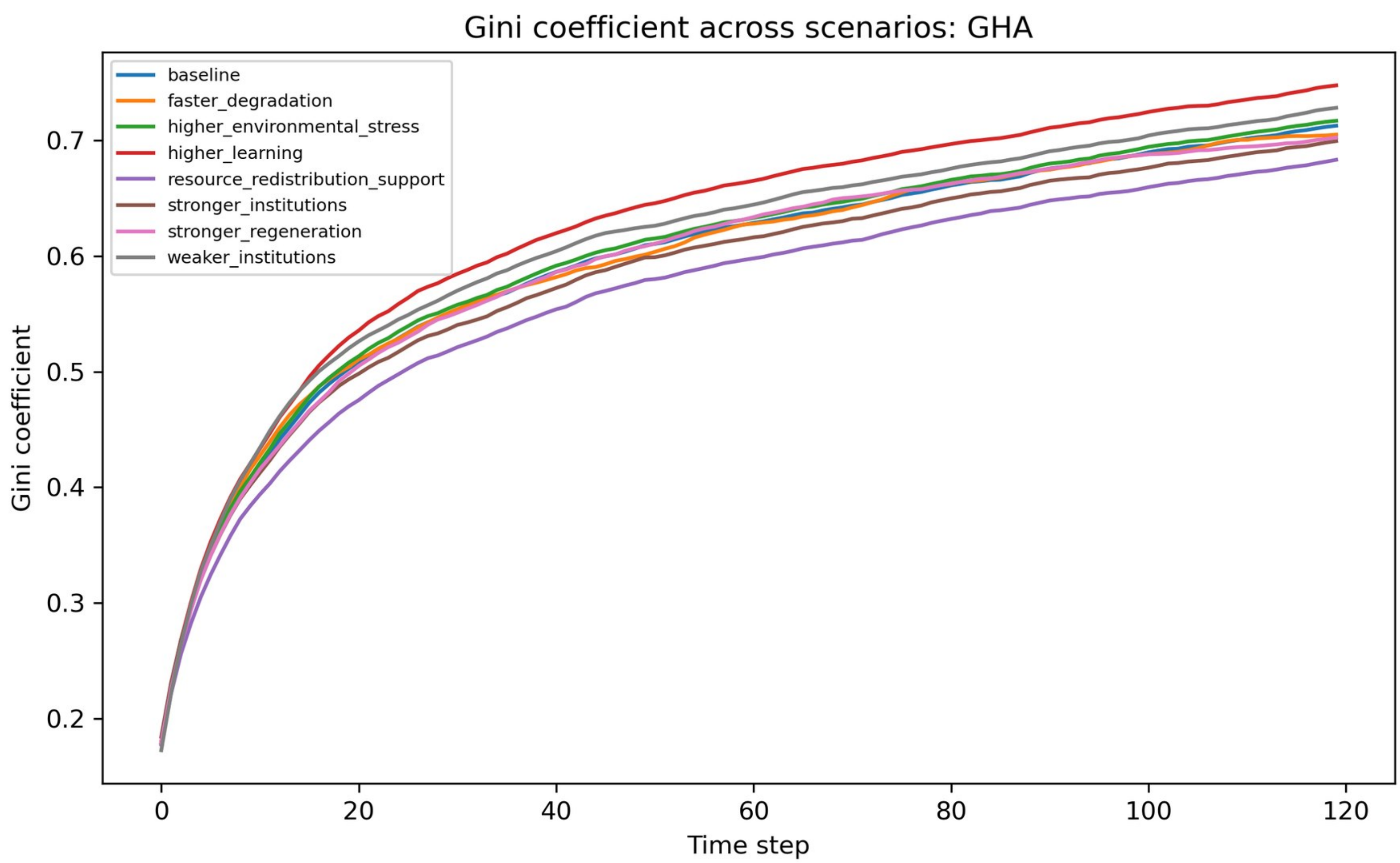

14. Ghana - Cooperation trajectory

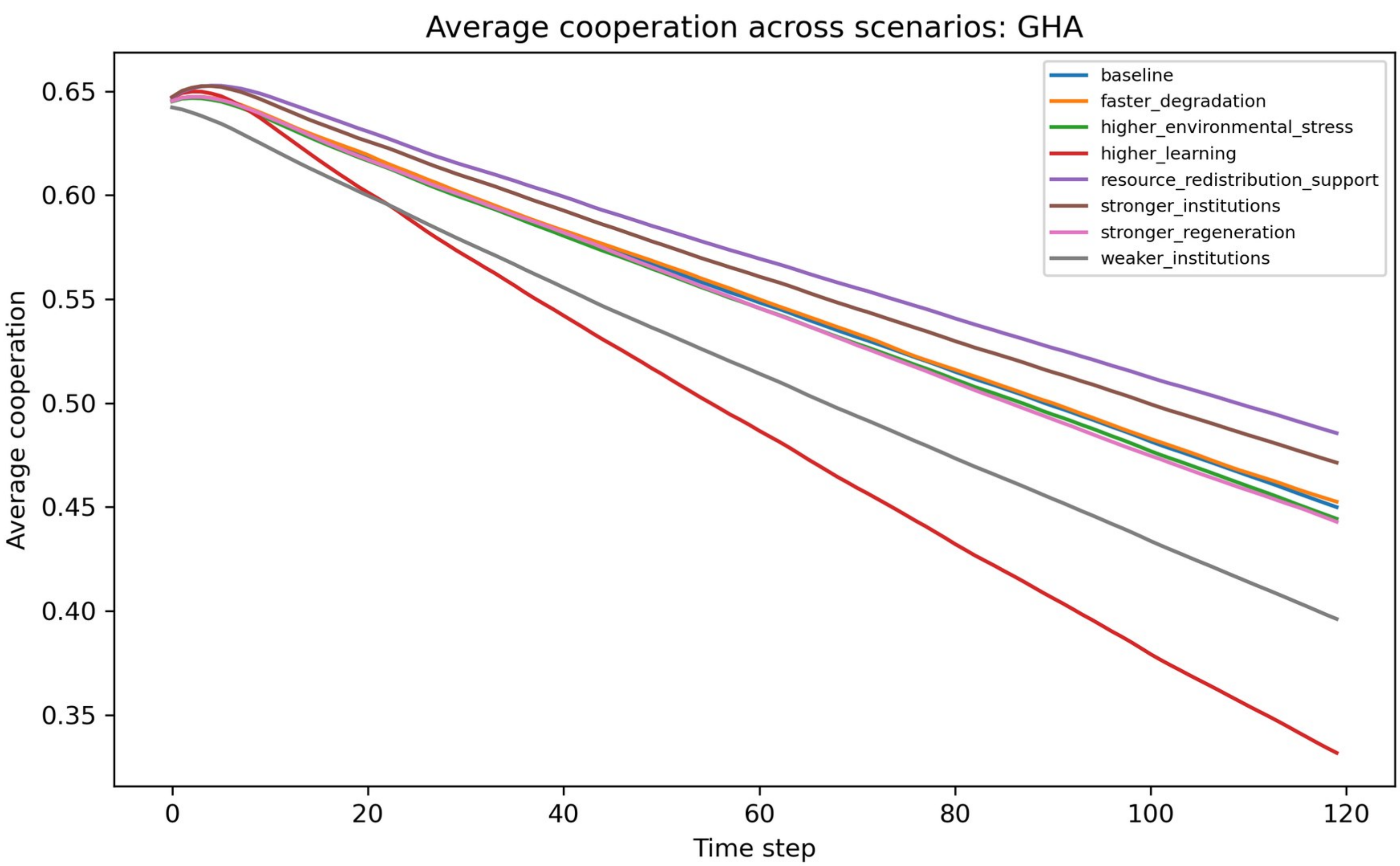

15. Ghana - Mean-resource trajectory

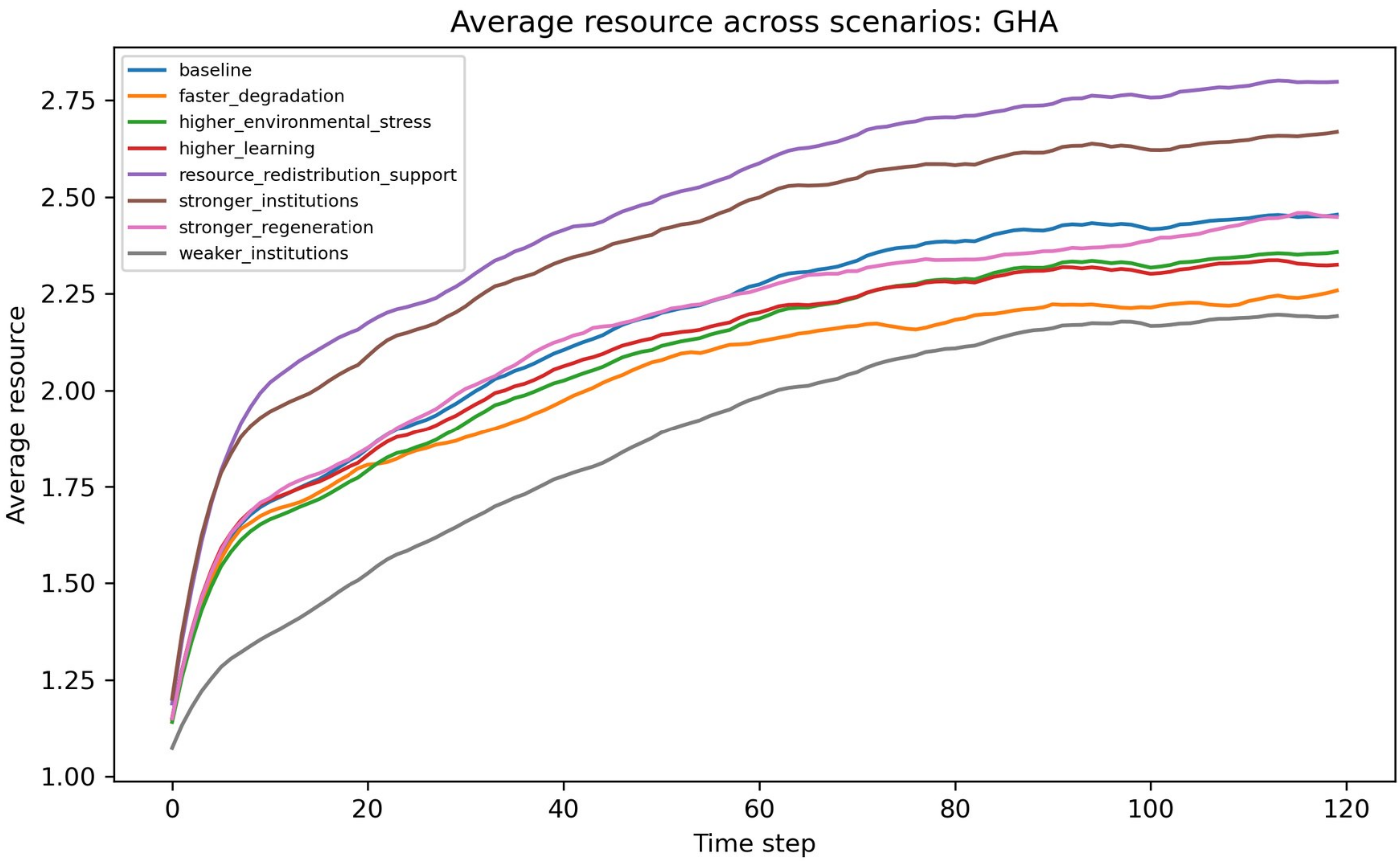

16. Kenya - Conflict-pressure trajectory

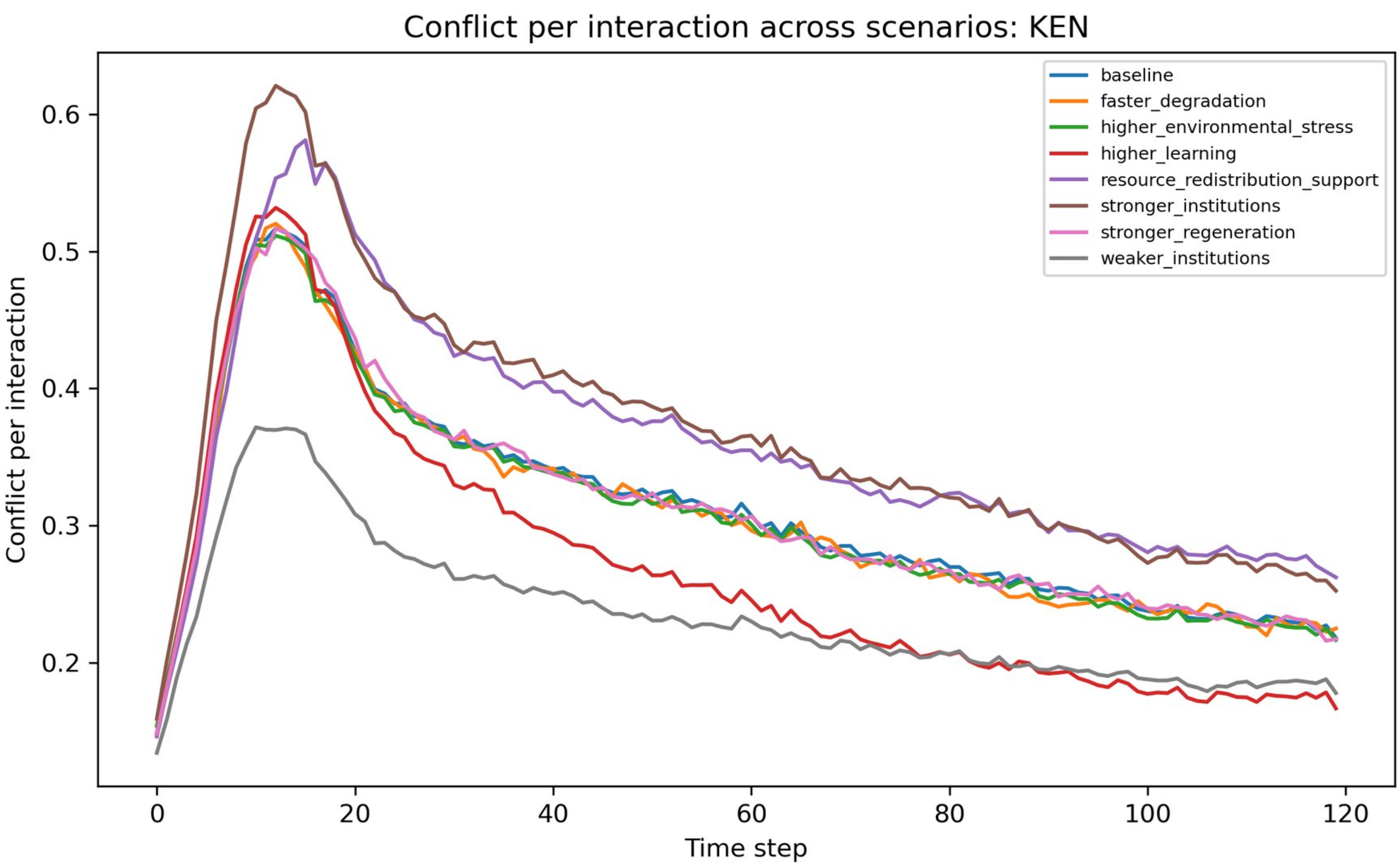

17. Kenya - Gini trajectory

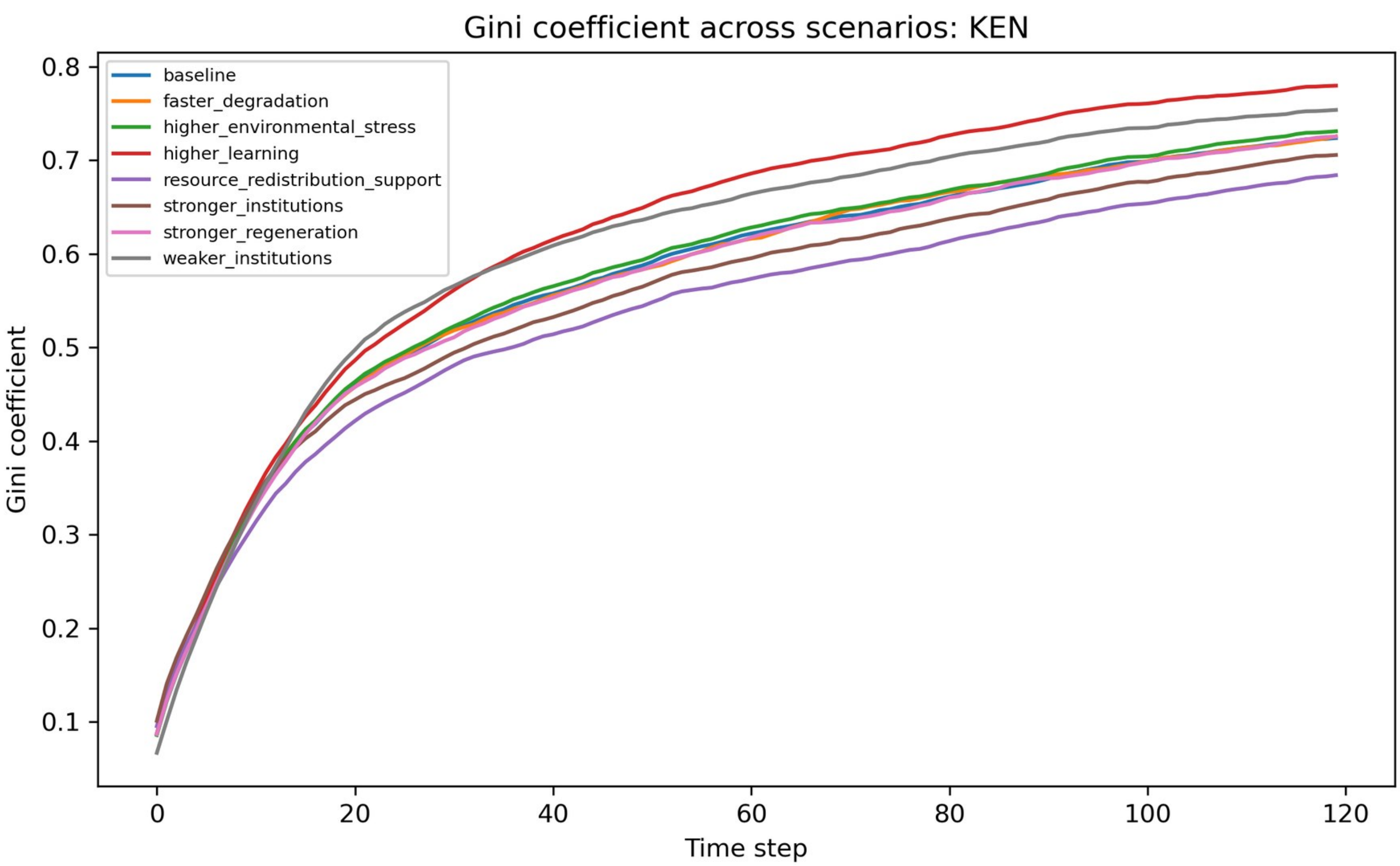

18. Kenya - Cooperation trajectory

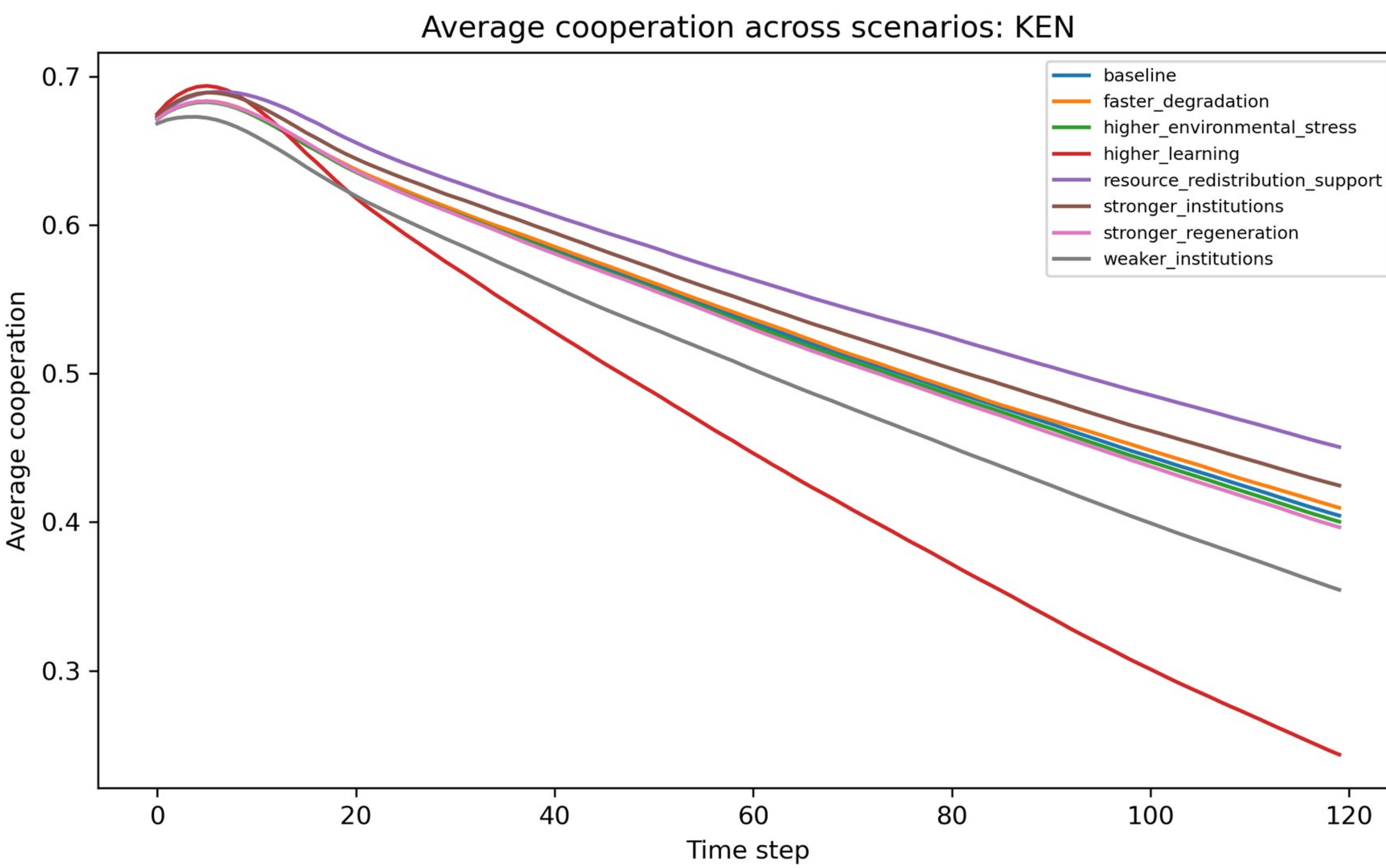

19. Kenya - Mean-resource trajectory

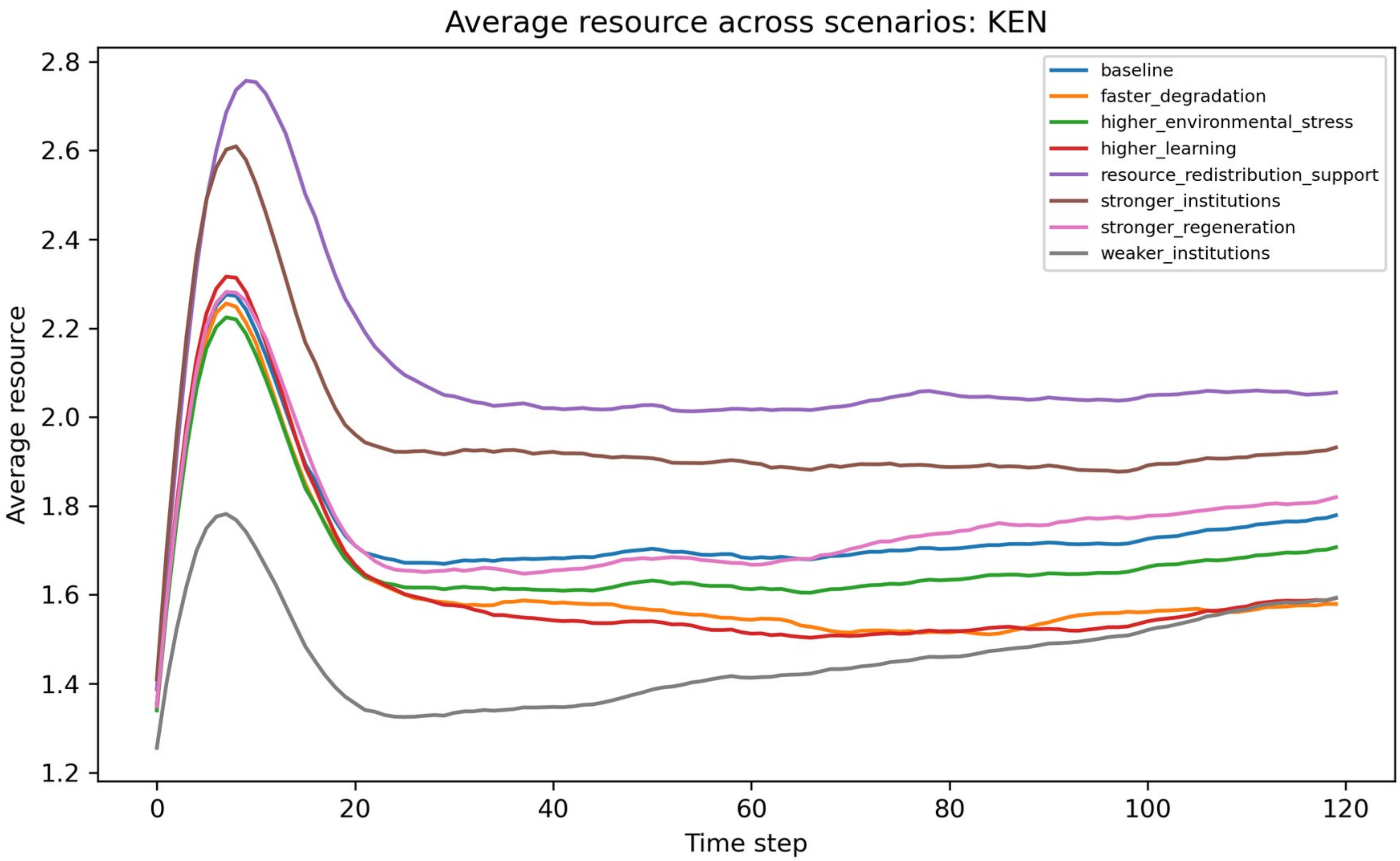

20. Madagascar - Conflict-pressure trajectory

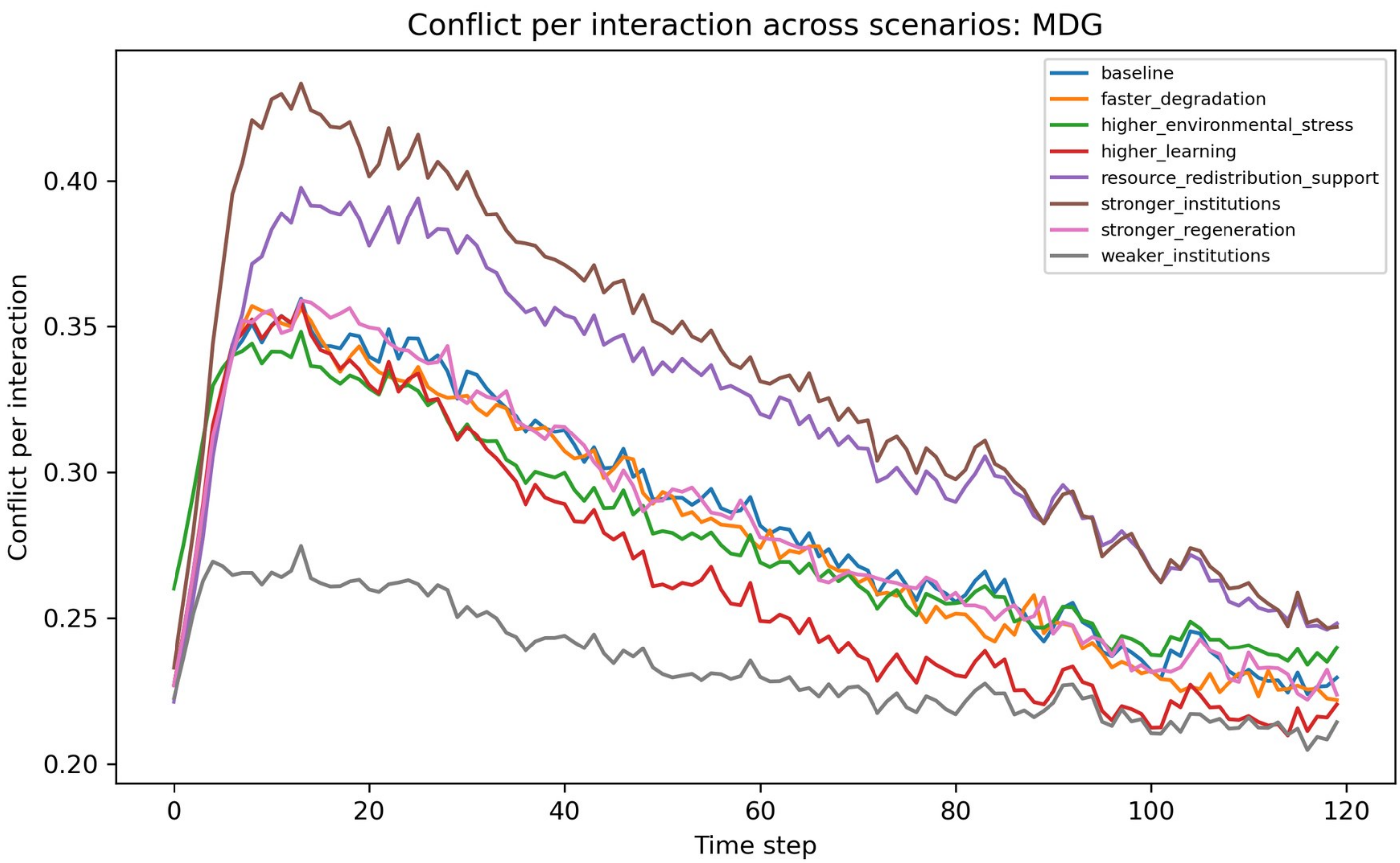

21. Madagascar - Gini trajectory

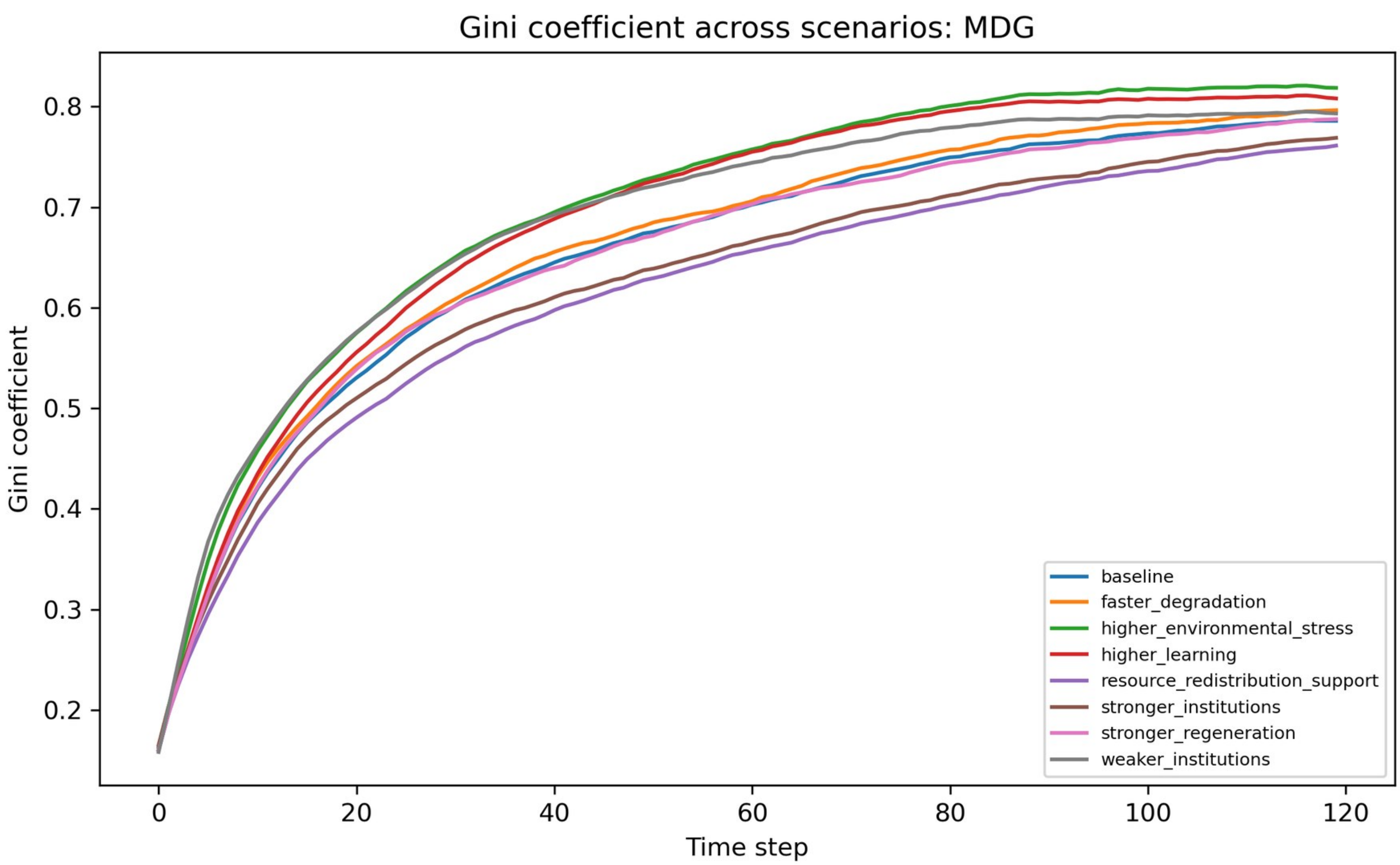

22. Madagascar - Cooperation trajectory

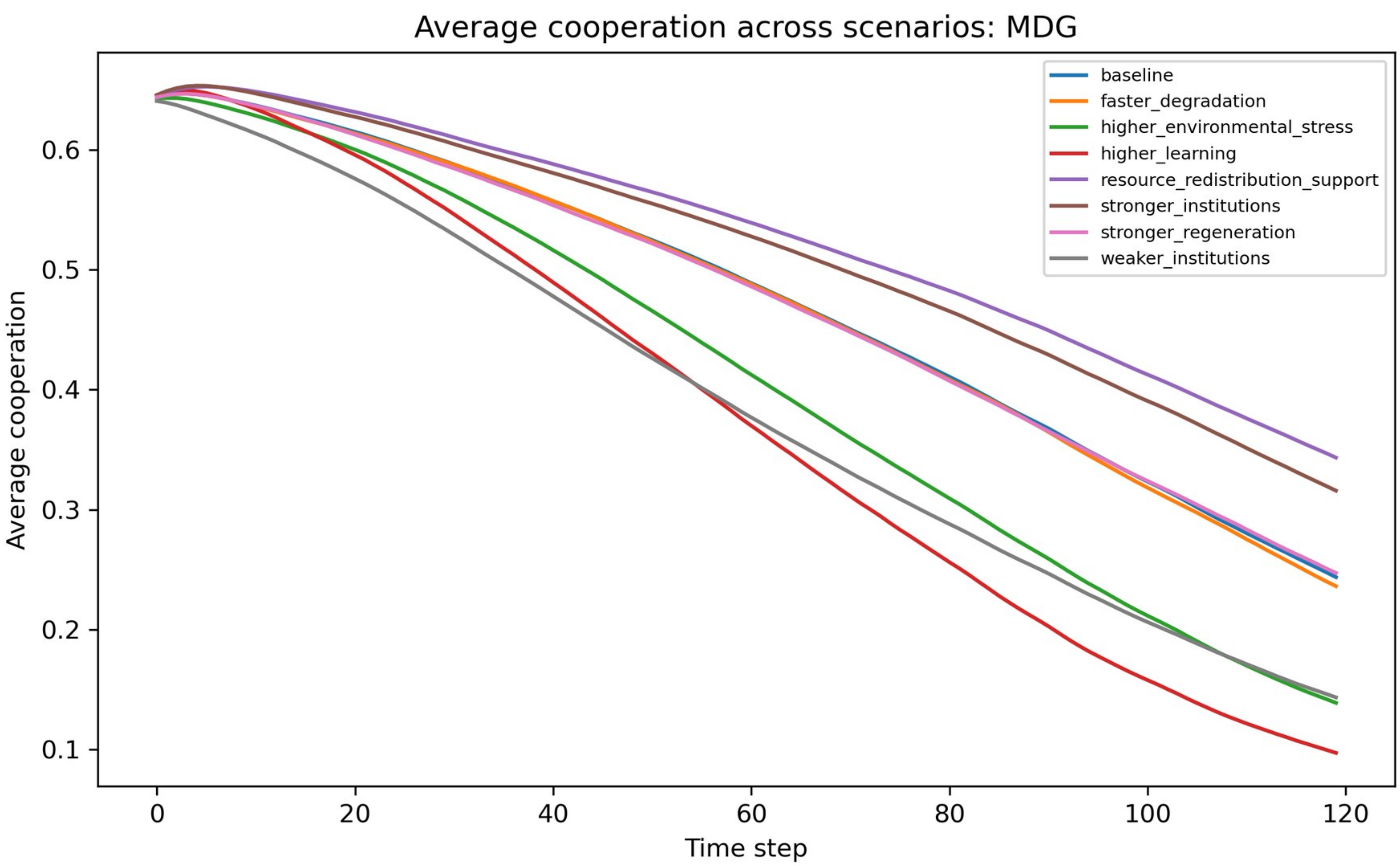

23. Madagascar - Mean-resource trajectory

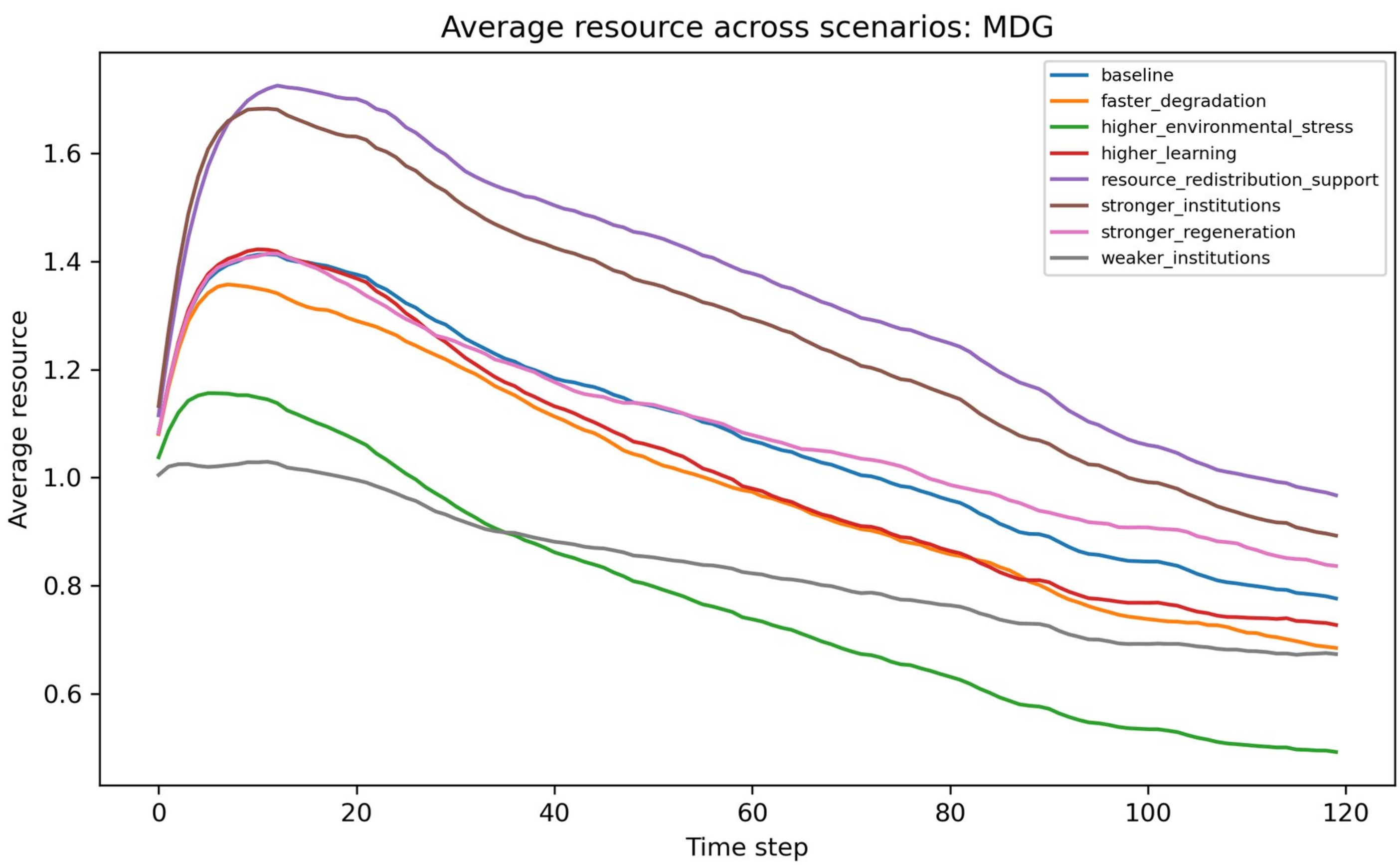

24. Nigeria - Conflict-pressure trajectory

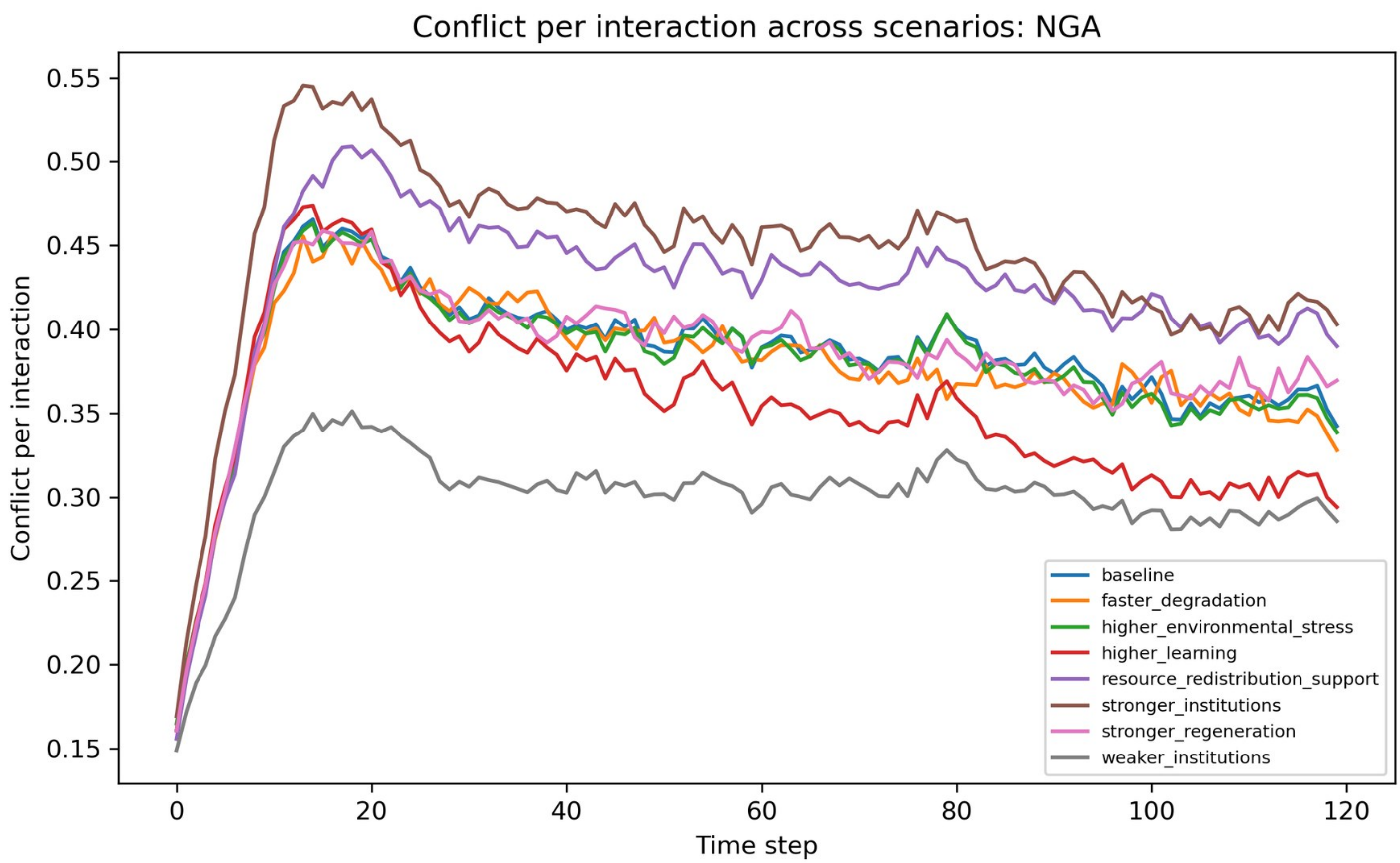

25. Nigeria - Gini trajectory

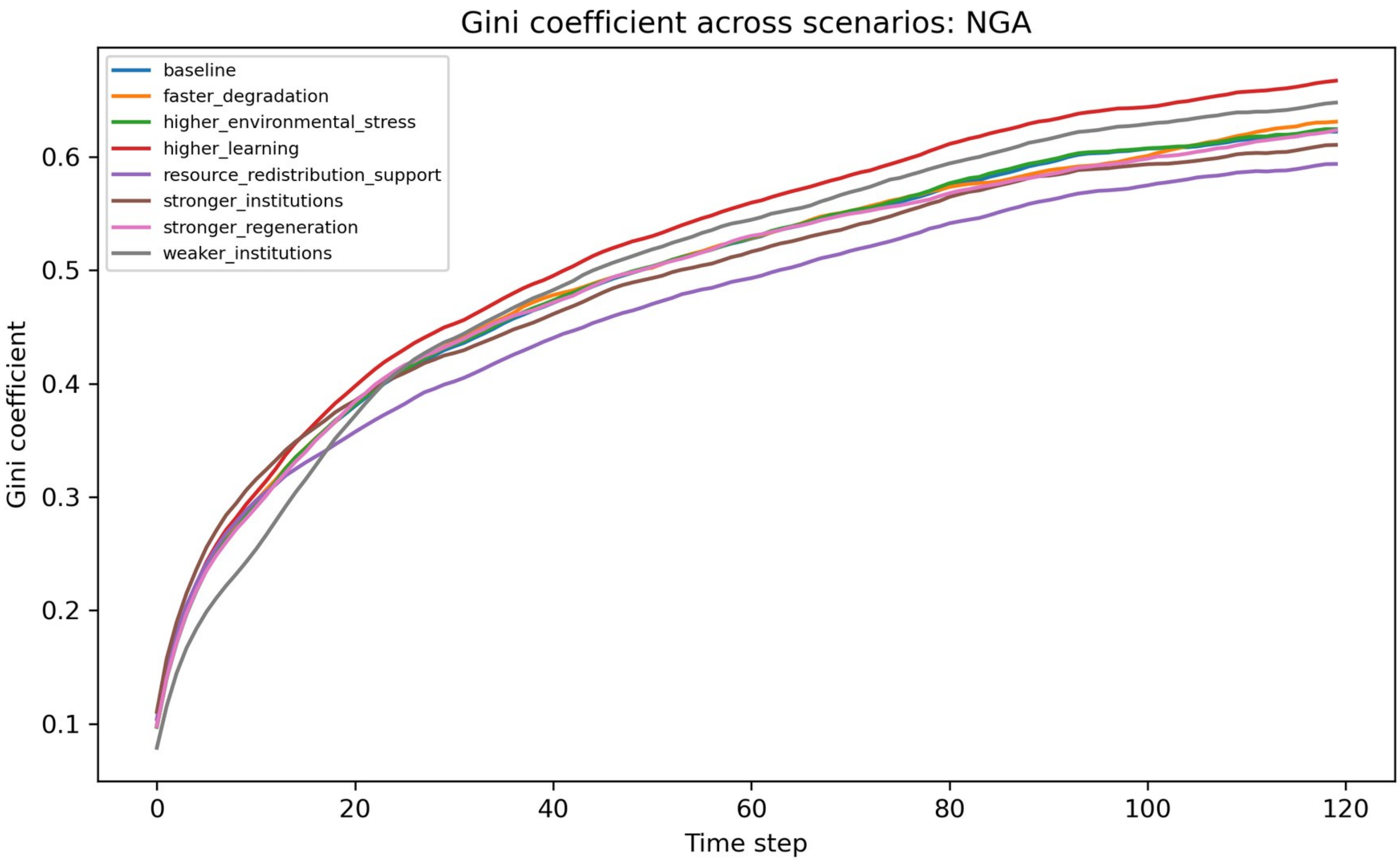

26. Nigeria - Cooperation trajectory

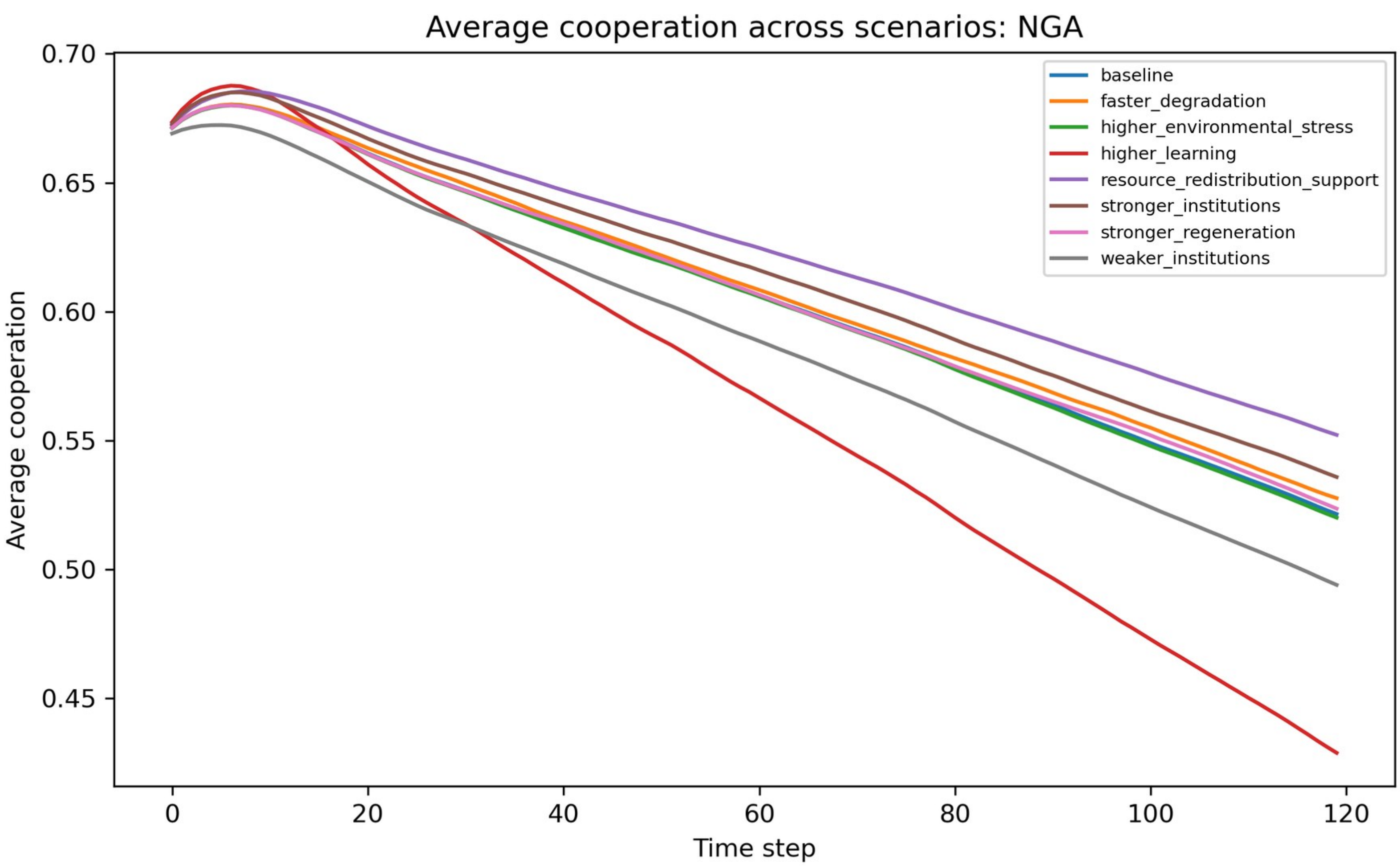

27. Nigeria - Mean-resource trajectory

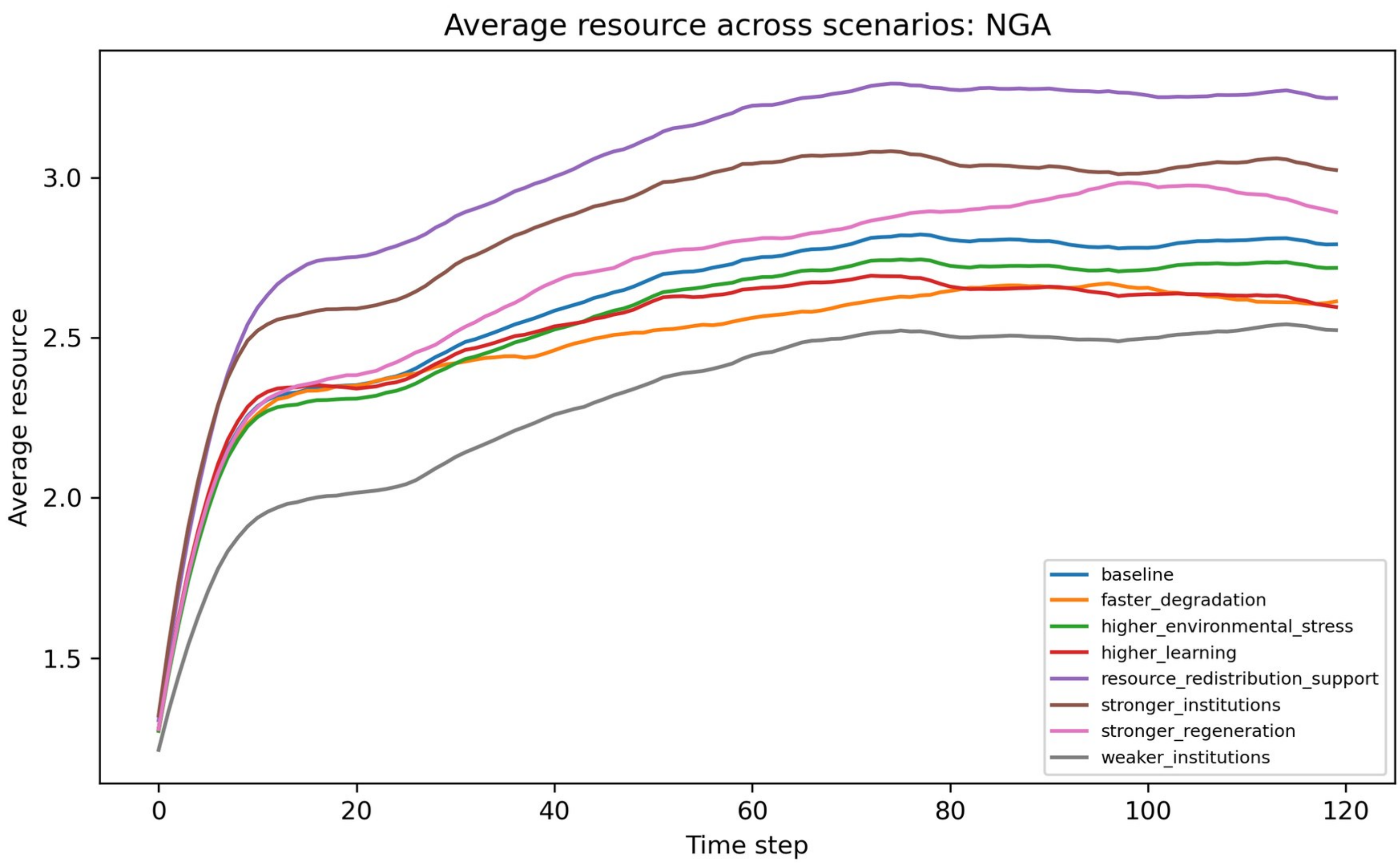

28. Somalia - Conflict-pressure trajectory

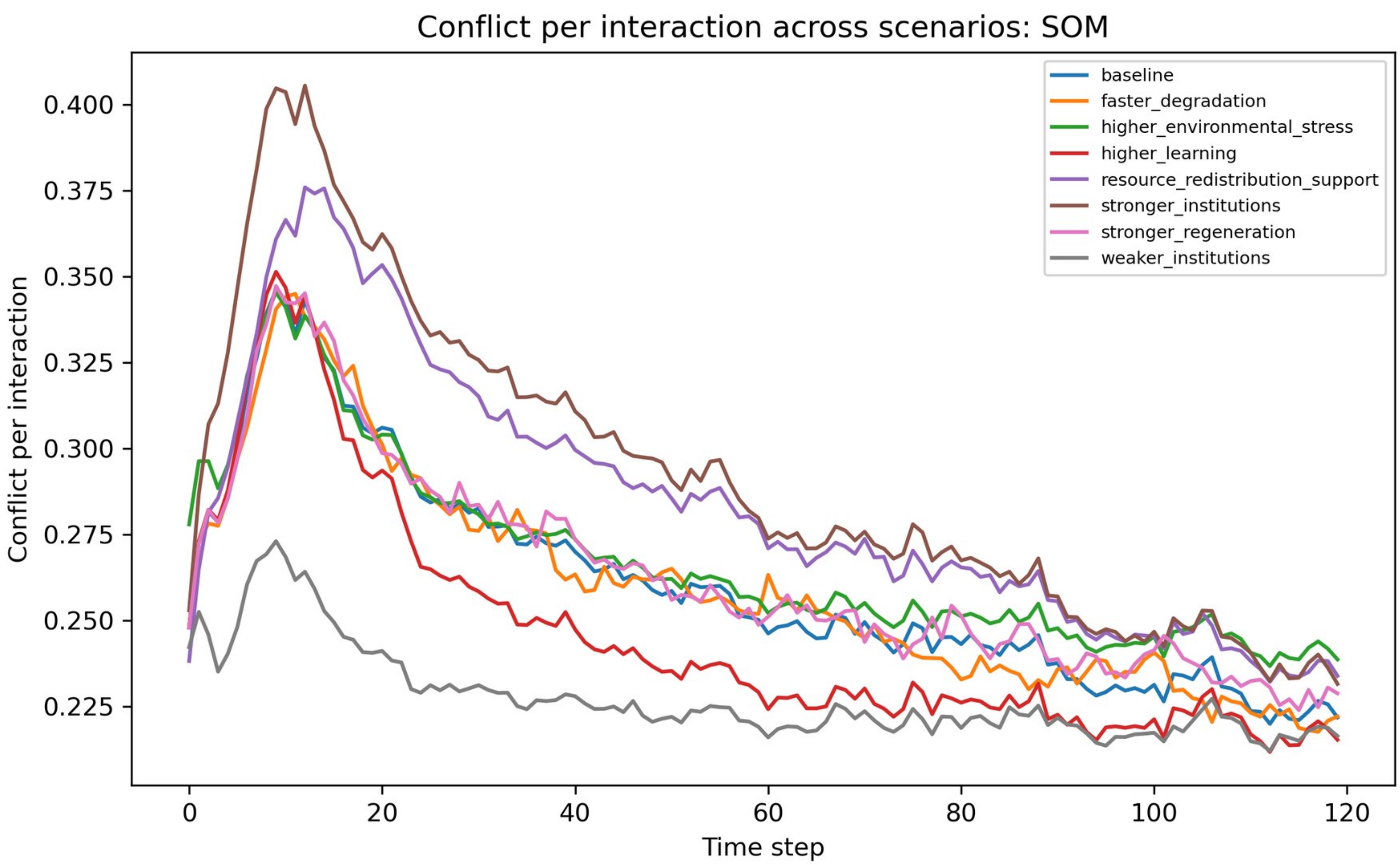

29. Somalia - Gini trajectory

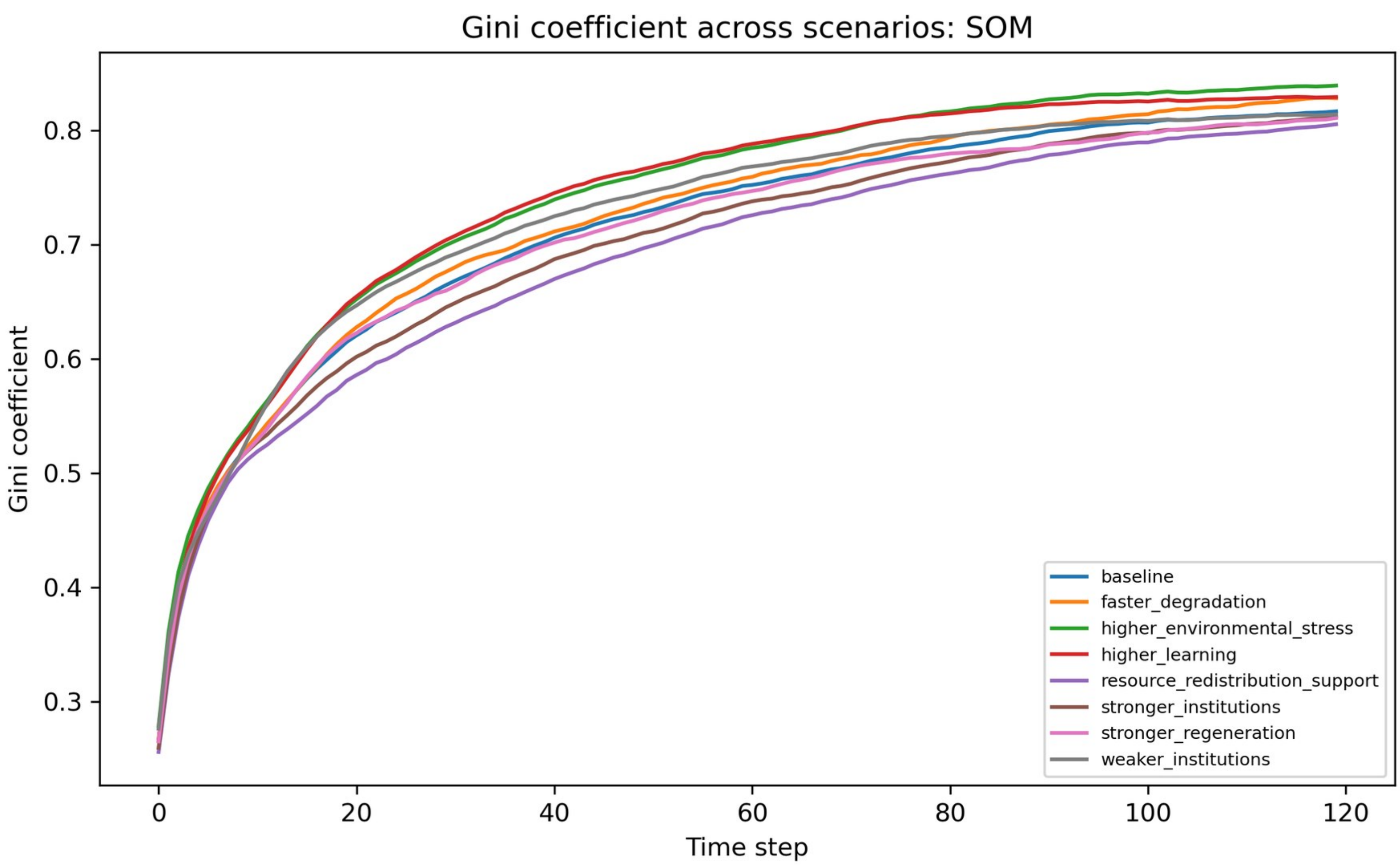

30. Somalia - Cooperation trajectory

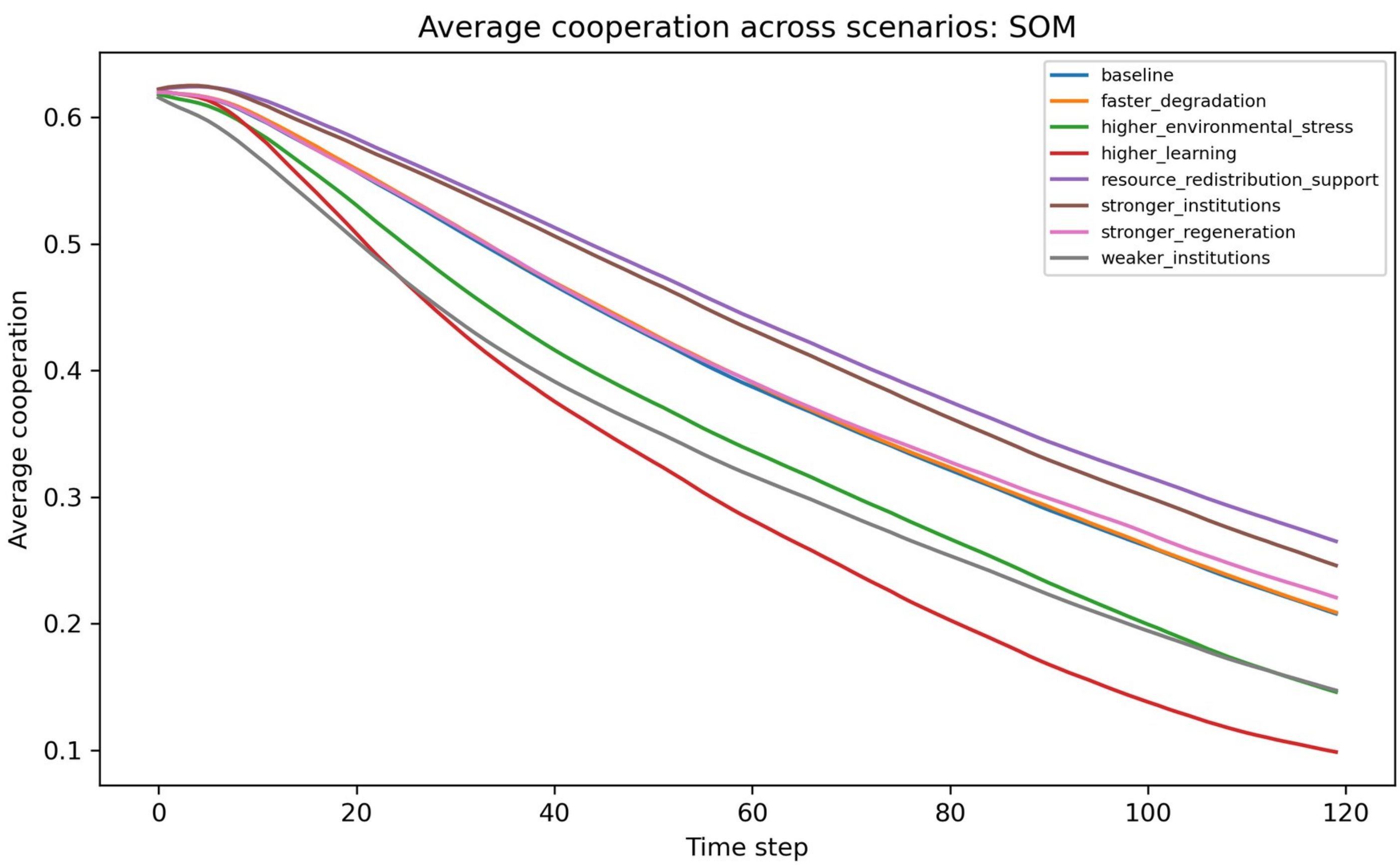

31. Somalia - Mean-resource trajectory

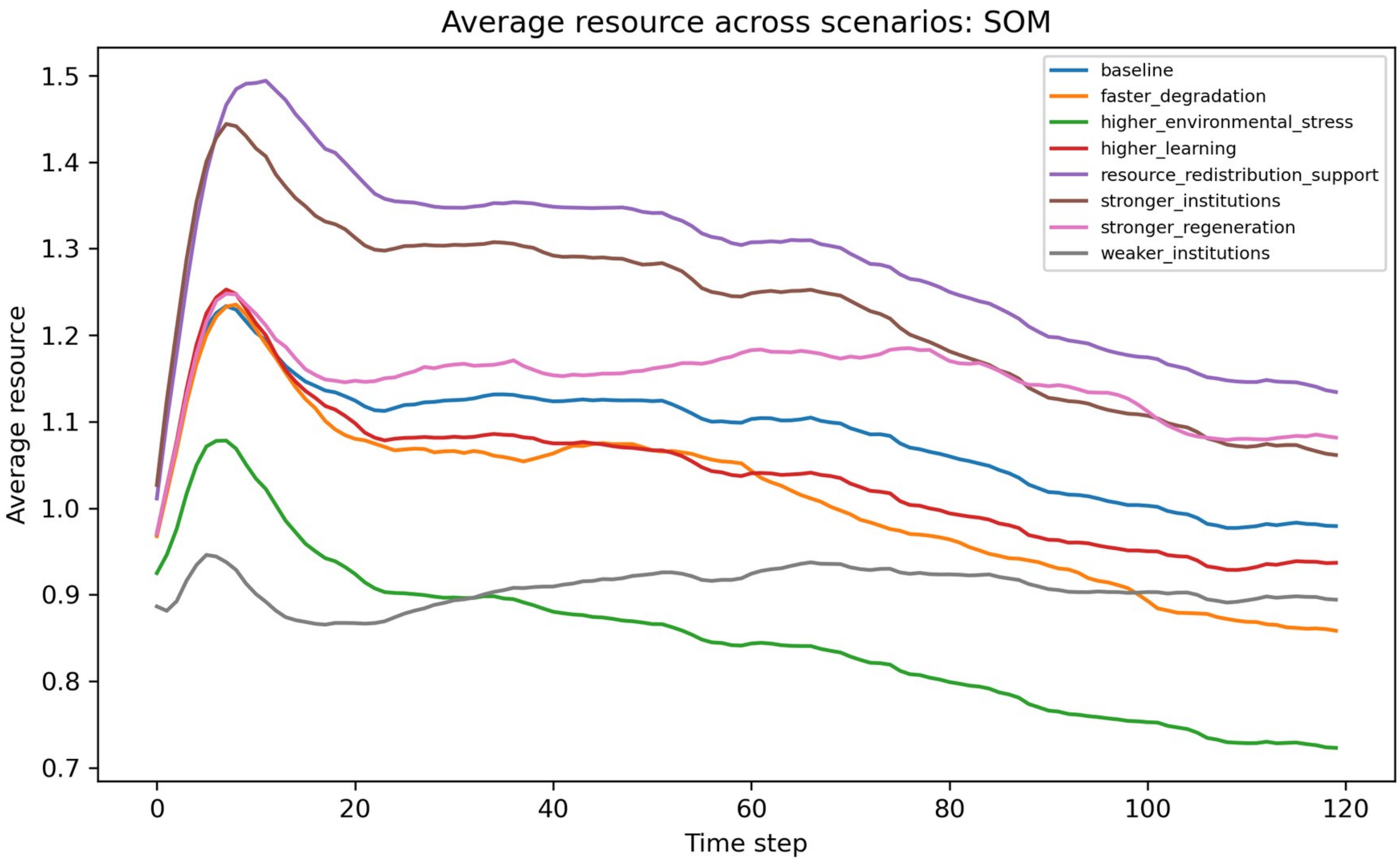

32. Tanzania - Conflict-pressure trajectory

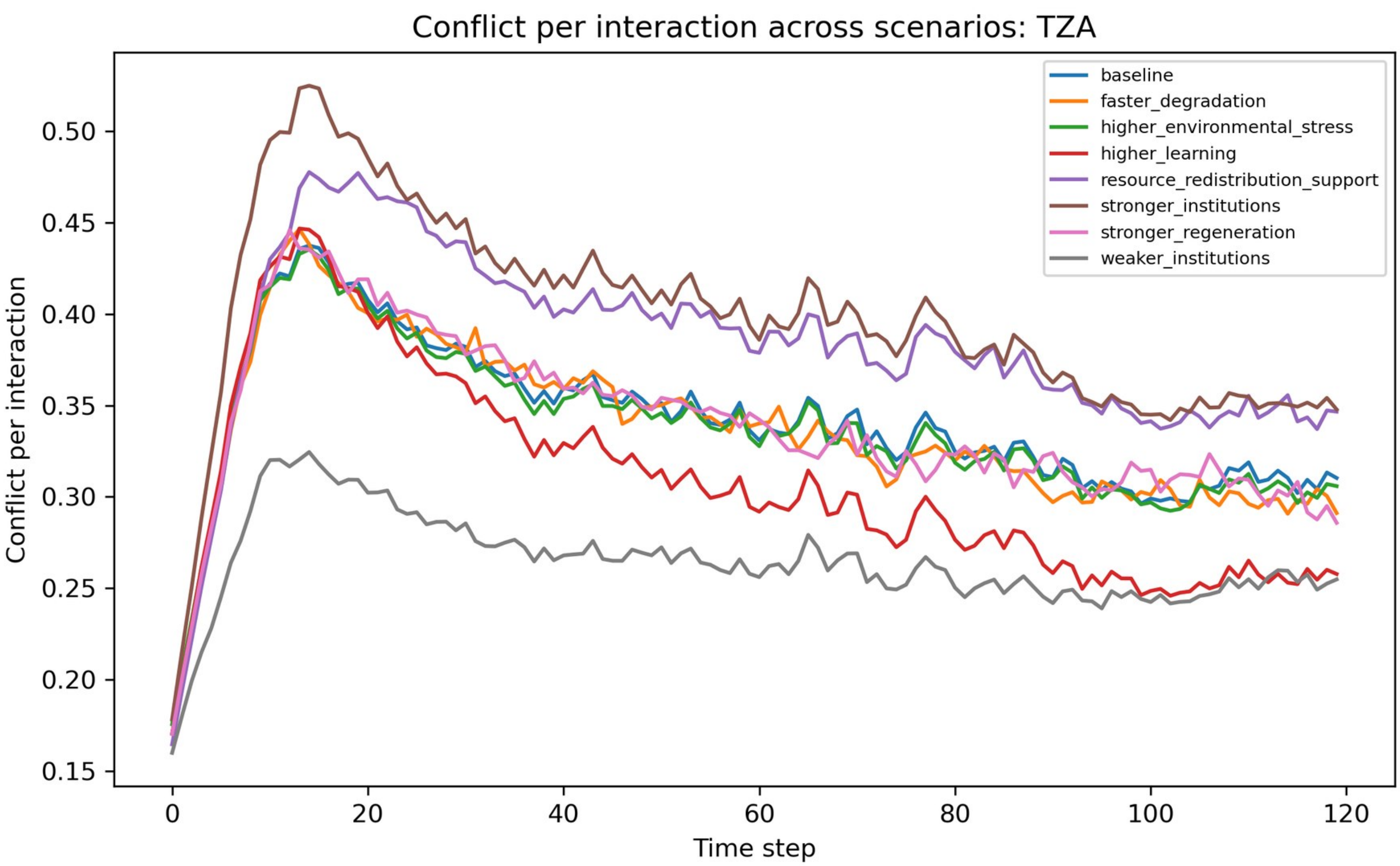

33. Tanzania - Gini trajectory

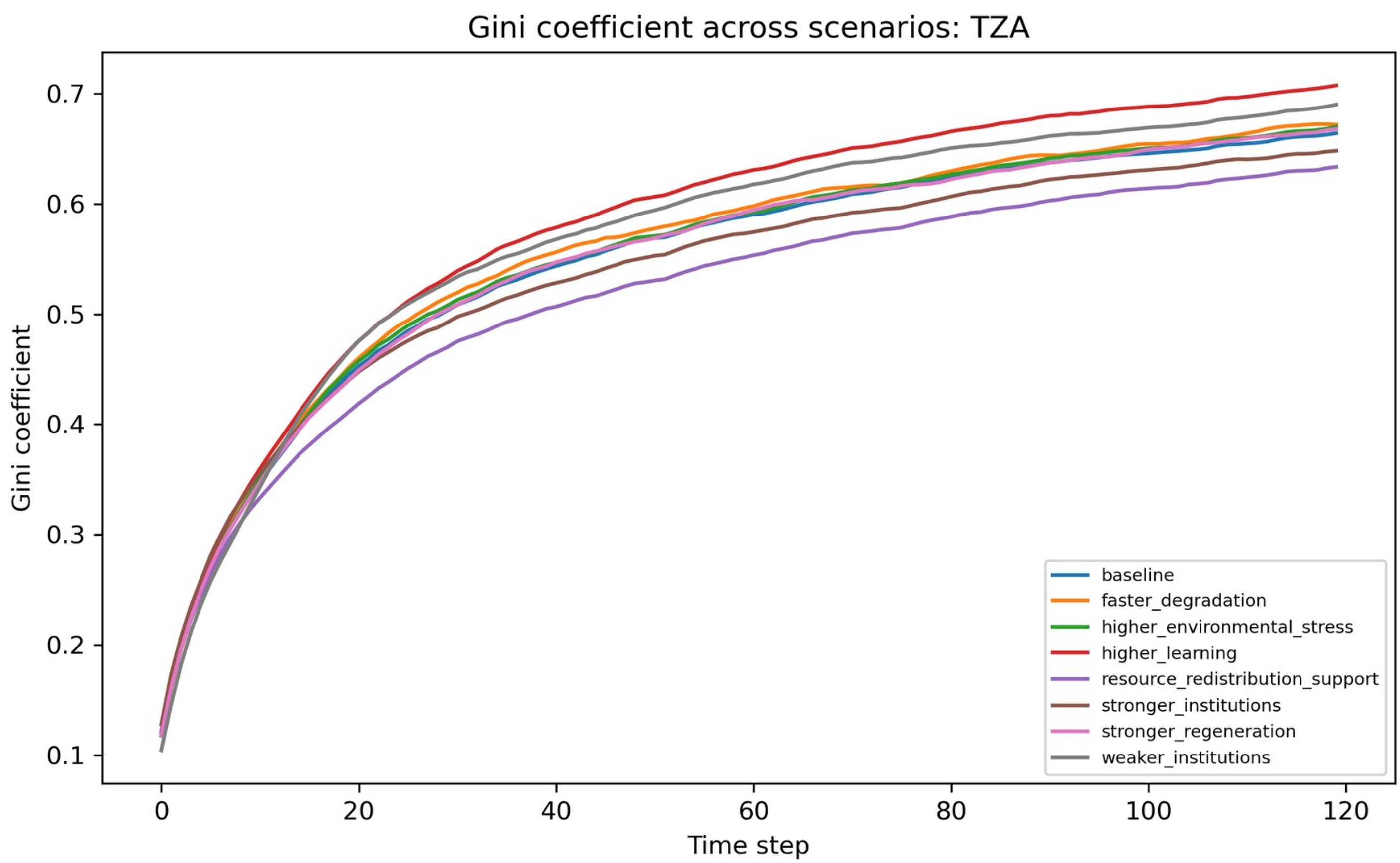

34. Tanzania - Cooperation trajectory

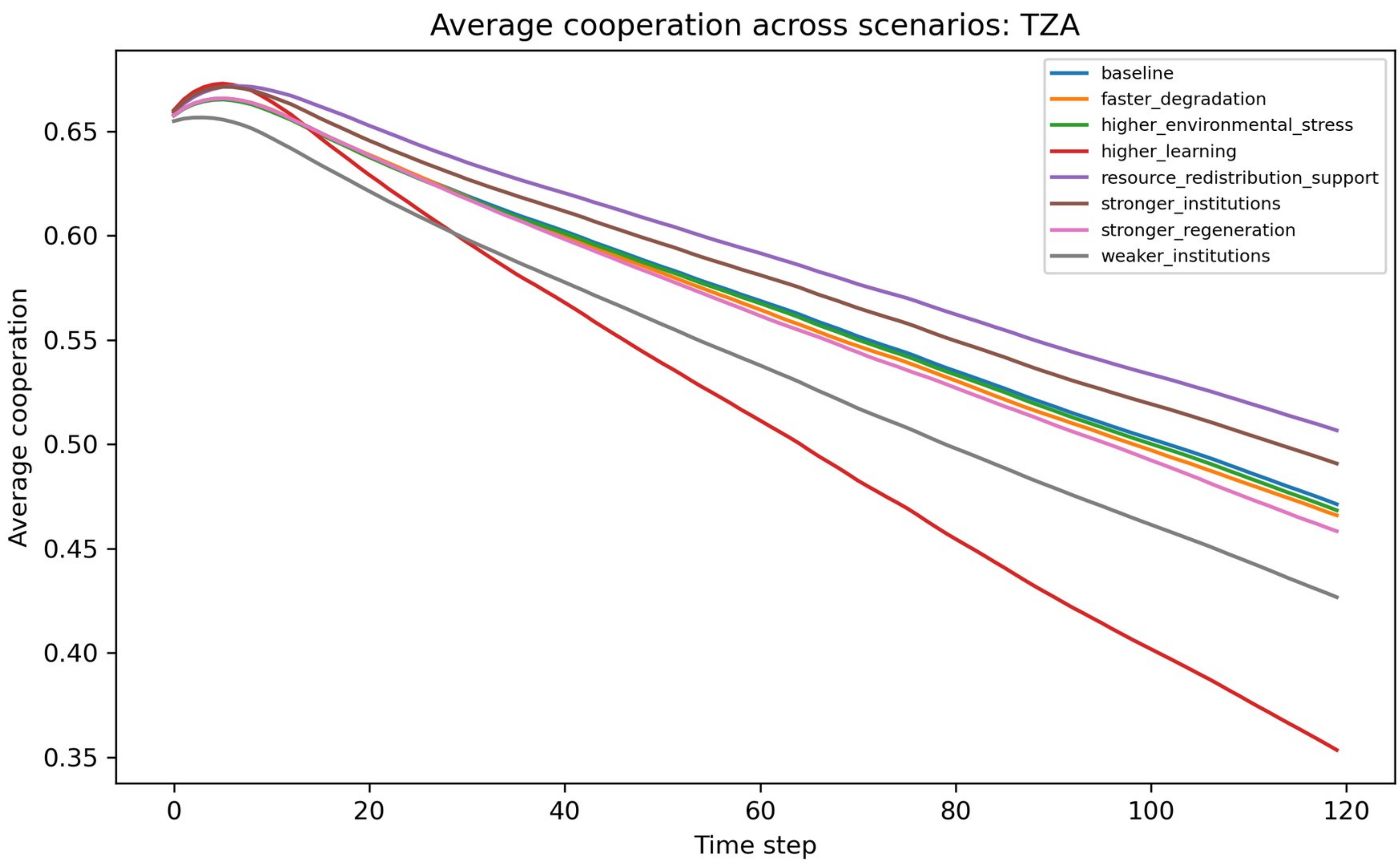

35. Tanzania - Mean-resource trajectory

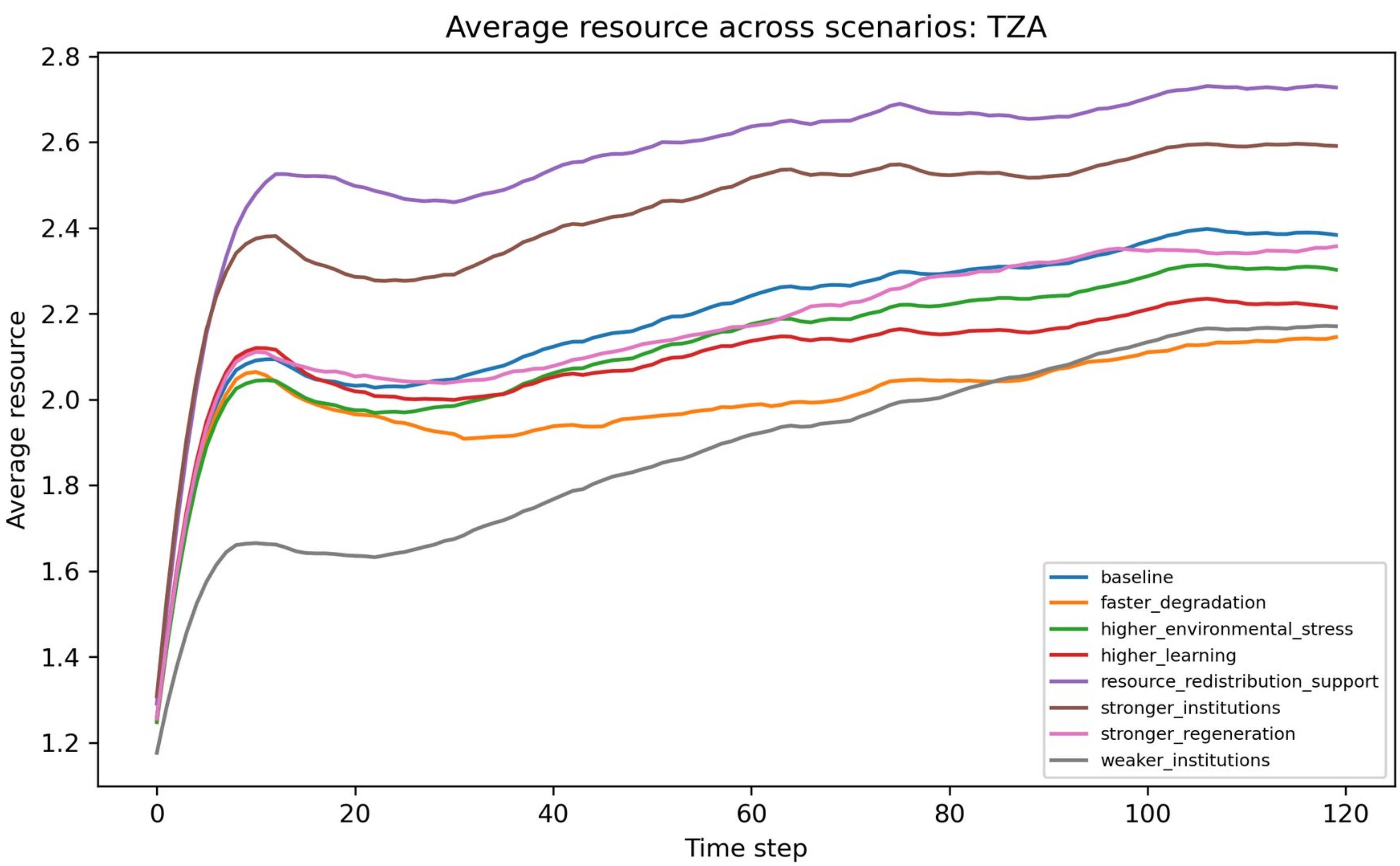

36. Uganda - Conflict-pressure trajectory

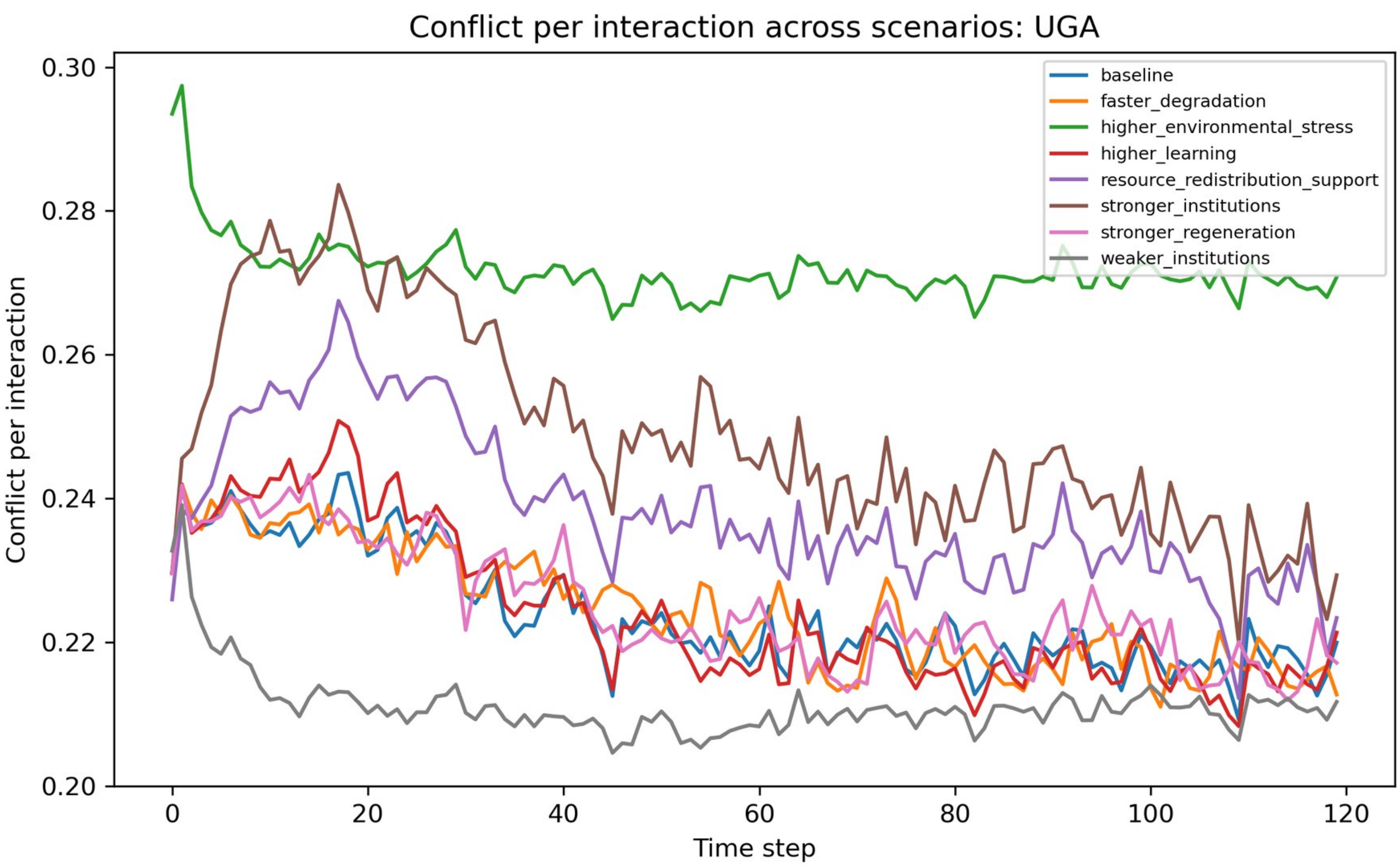

37. Uganda - Gini trajectory

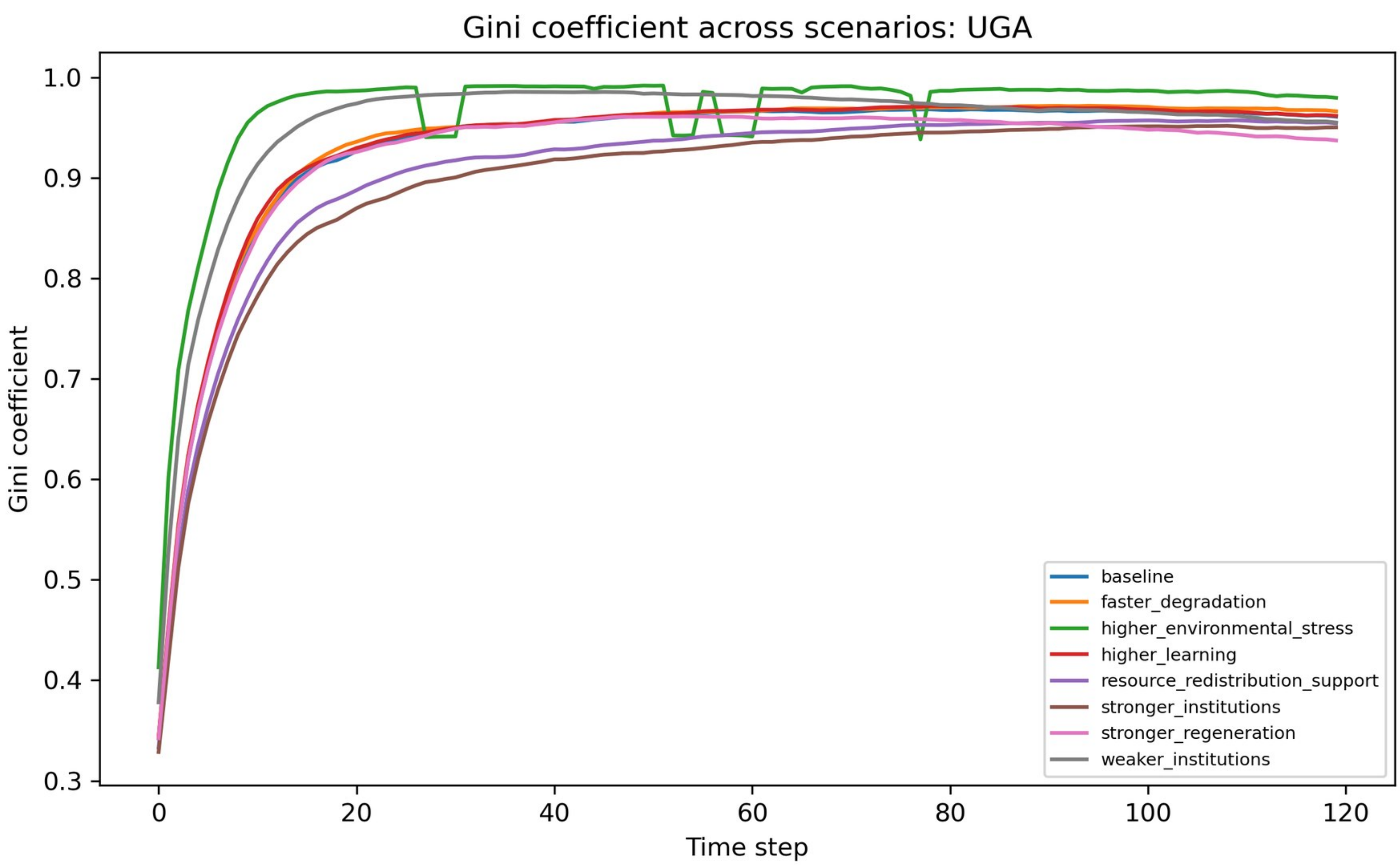

38. Uganda - Cooperation trajectory

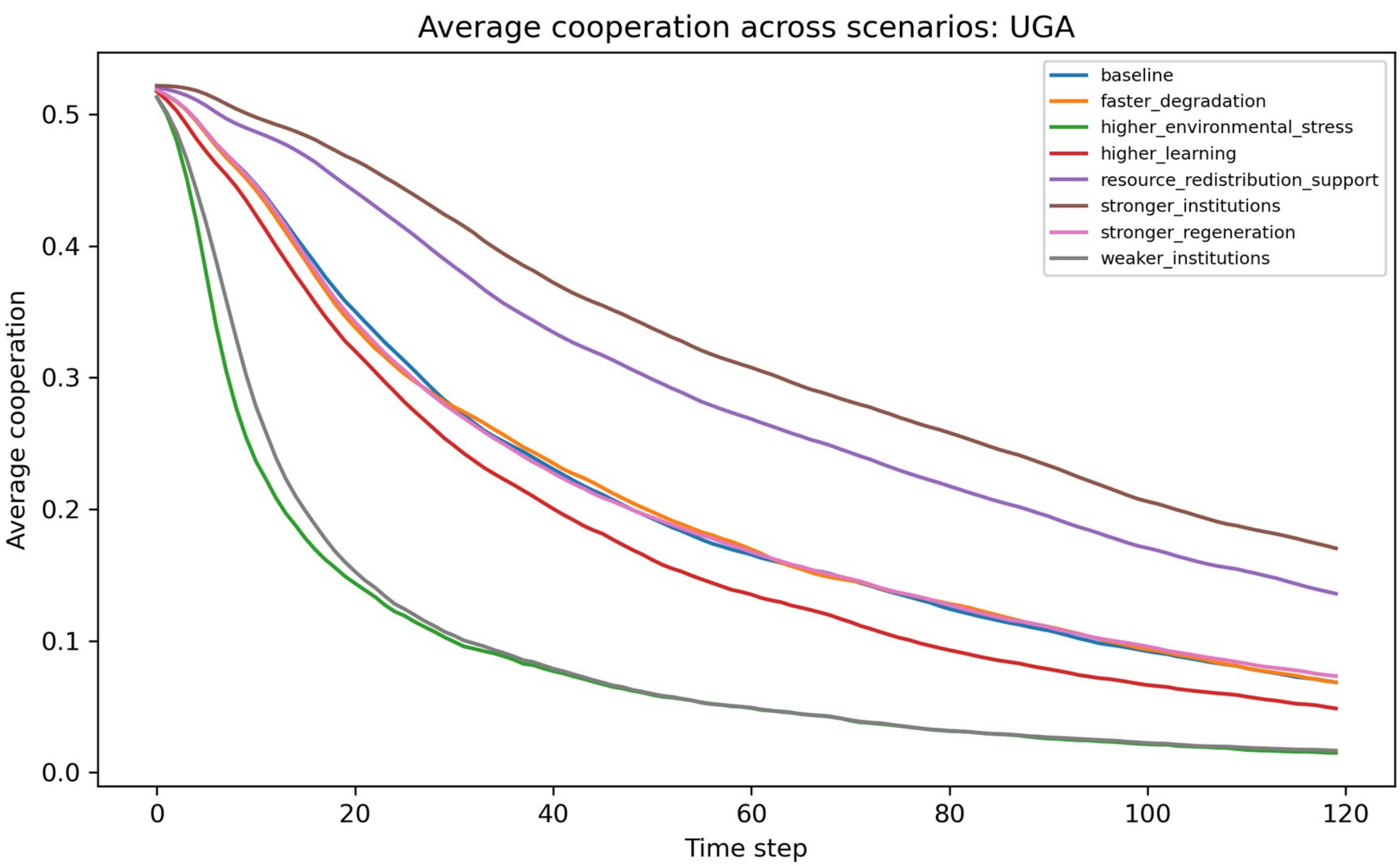

39. Uganda - Mean-resource trajectory

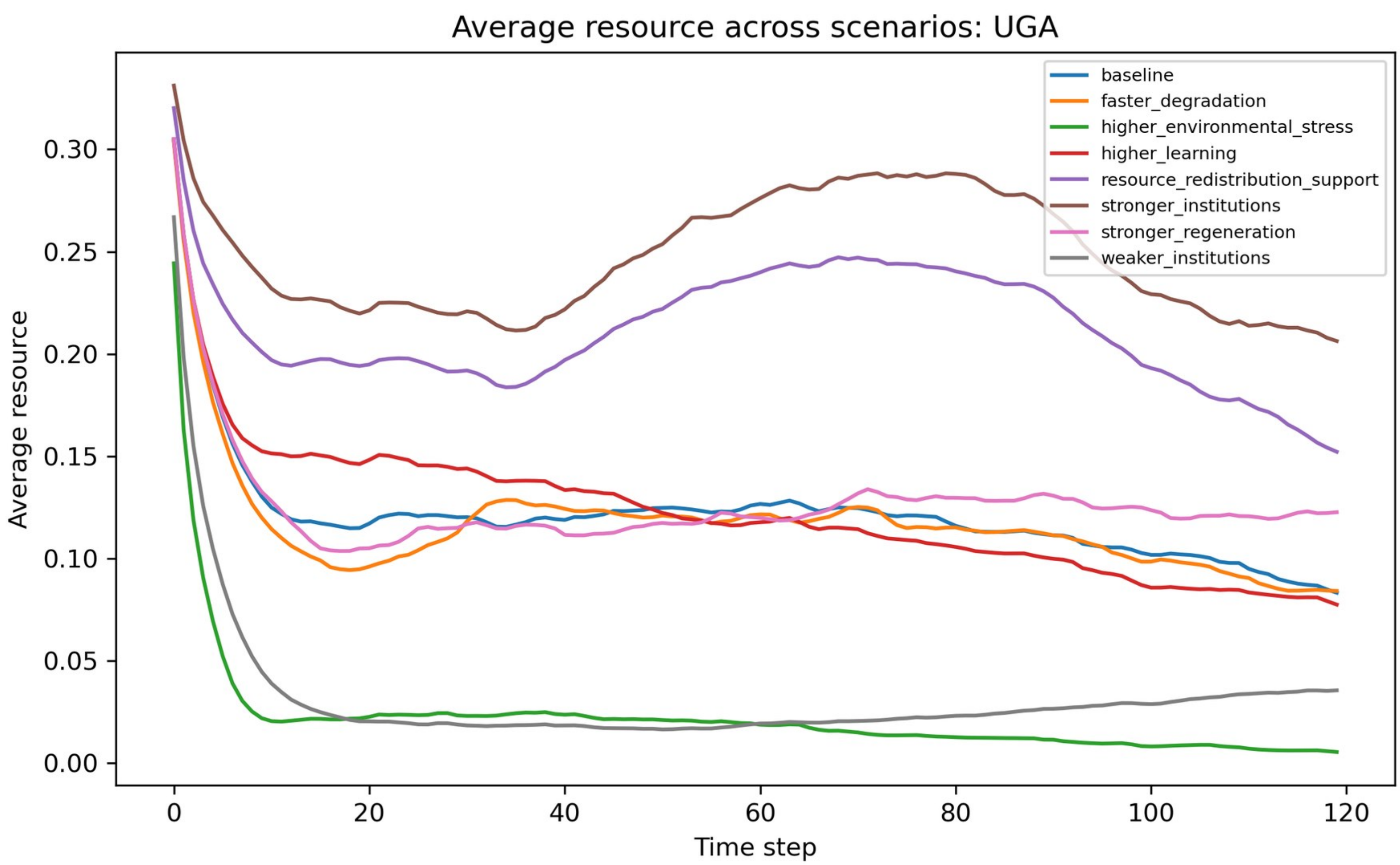

Supplement: Supplementary file 2 [file pone.0357283.s005.pdf]
